# Supplementary material for: Halide-assisted differential growth of chiral nanoparticles with threefold rotational symmetry
Source: Nat Commun. 2023 Jun 24;14:3783. doi: 10.1038/s41467-023-39456-8 (PMC10290678; doi:10.1038/s41467-023-39456-8)
Supplement: Supplementary file 1 — Supplementary Information File [file 41467_2023_39456_MOESM1_ESM.pdf]

Supplementary information

Halide-assisted differential growth of chiral nanoparticles with threefold rotational symmetry

Authors: Jiapeng Zheng[1,2,8], Christina Boukouvala[3,4,8], George Lewis[3,4,8], Yicong Ma[5], Yang Chen[1], Emilie Ringe[3,4]*, Lei Shao[6]*, Zhifeng Huang[7] & Jianfang Wang[1,2]*

Affiliations:

[1]Department of Physics, The Chinese University of Hong Kong, Shatin, Hong Kong SAR, China.

[2]Shenzhen Research Institute, The Chinese University of Hong Kong, Shenzhen 518057, China.

[3]Department of Materials Science and Metallurgy, University of Cambridge, Cambridge CB3 0FS, United Kingdom.

[4]Department of Earth Sciences, University of Cambridge, Cambridge CB2 3EQ, United Kingdom.

[5]Department of Physics, Hong Kong Baptist University, Kowloon Tong, Hong Kong SAR, China.

[6]State Key Laboratory of Optoelectronic Materials and Technologies, Guangdong Province Key Laboratory of Display Material and Technology, School of Electronics and Information Technology, Sun Yat-sen University, Guangzhou 510275, China

[7]Department of Chemistry, The Chinese University of Hong Kong, Shatin, Hong Kong SAR, China

[8]These authors contributed equally to this work.

*Corresponding authors. E-mail: jfwang@phy.cuhk.edu.hk (J.F.W.); er407@cam.ac.uk (E.R.); shaolei5@mail.sysu.edu.cn (L.S.)

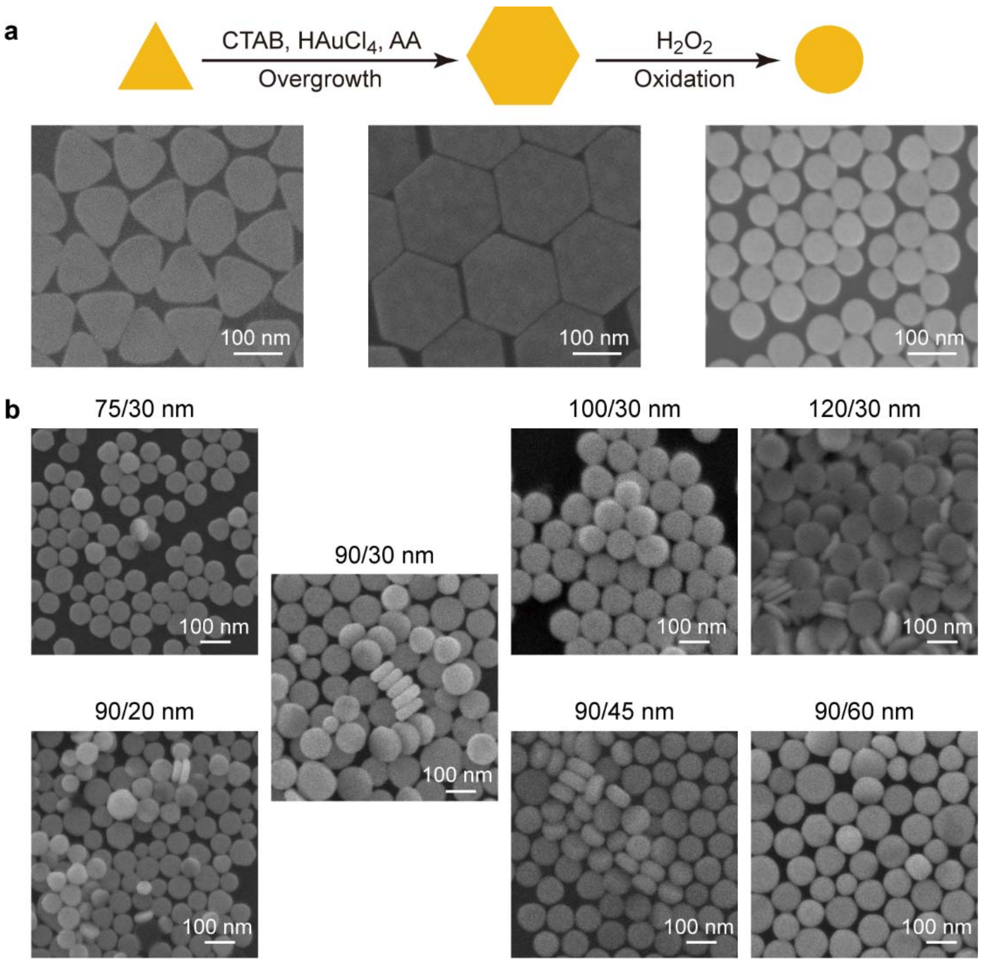

Supplementary Fig. 1 | Circular Au nanodisk seeds. a, Schematic illustrating the synthesis route. Triangular Au nanoplates were first produced and then grown into hexagonal nanoplates. Circular Au nanodisks were obtained through anisotropic oxidation of the hexagonal Au nanoplates. The structural change was observed from the SEM images. **b**, SEM images of the Au nanodisks of different dimensions. The sizes and LSPR peak wavelengths of the nanodisk samples were summarized in Supplementary Table 1. For example, the dimension of 75/30 nm means that the Au nanodisks have an average diameter of 74.8 ± 8.4 nm and an average thickness of 29.5 ± 4.3 nm.

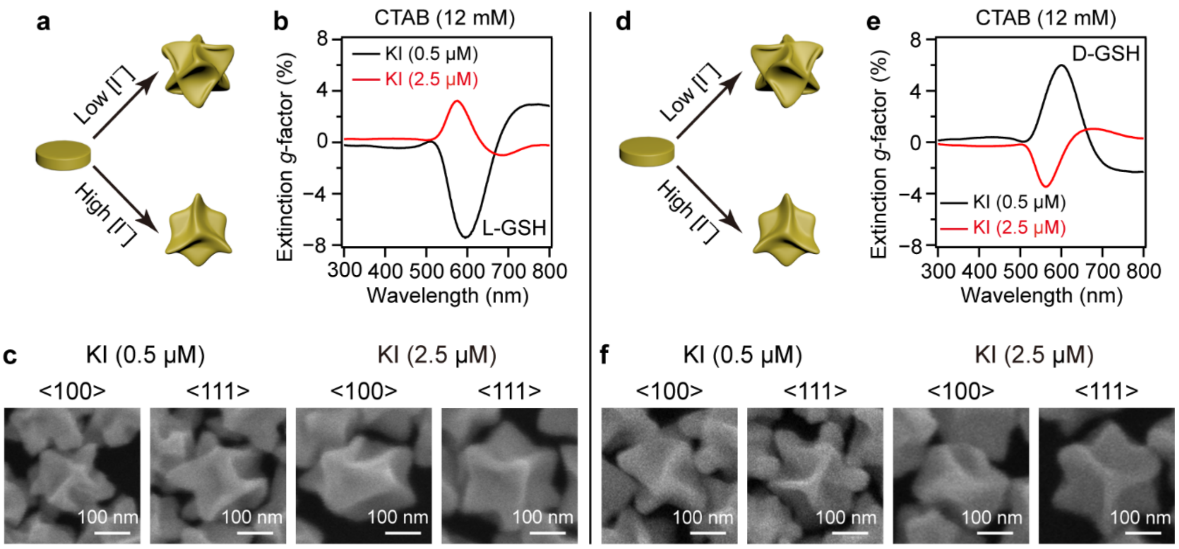

Supplementary Fig. 2 | Halide-assisted differential growth (HADG) on the 90/30 nm anisotropic nanodisks. a–**c**, Chiral growth guided by L-glutathione (GSH). **d**–**f**, Chiral growth guided by D-GSH. The morphology and the chiroptical properties of the obtained chiral nanocrystals can be controlled by the growth rates along the <100> and <111> directions. We used CTAB and KI to control the growth rates along the different directions, respectively. The employment of KI at low and high concentrations results in the formation of 432 helicoid III and triskelion-shaped Au nanocrystals, respectively (**a**,**d**). These two types of chiral nanocrystals present opposite chiroptical responses (**b**,**e**) and show large structural differences along the <100> and <111> directions (**c**,**f**). All the scale bars in (**c**,**f**) are the same. The large-area SEM images of these chiral nanocrystals are shown in Supplementary Figs. 3, 4. Source data are provided as a Source Data file.

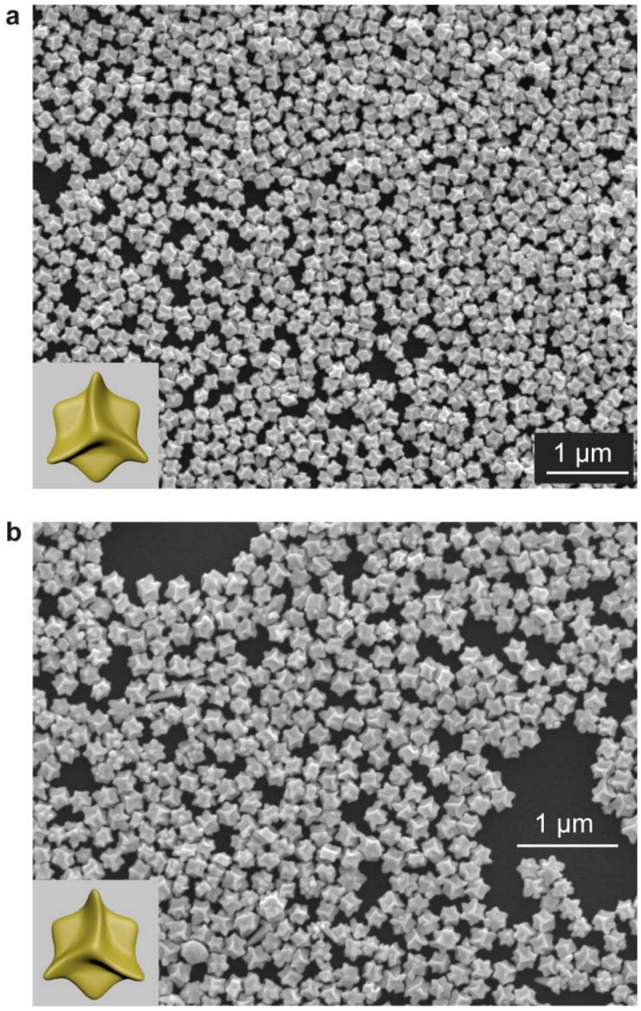

Supplementary Fig. 3 | Large-area SEM images. (**a**) L- and (**b**) D-nanotriskelions.

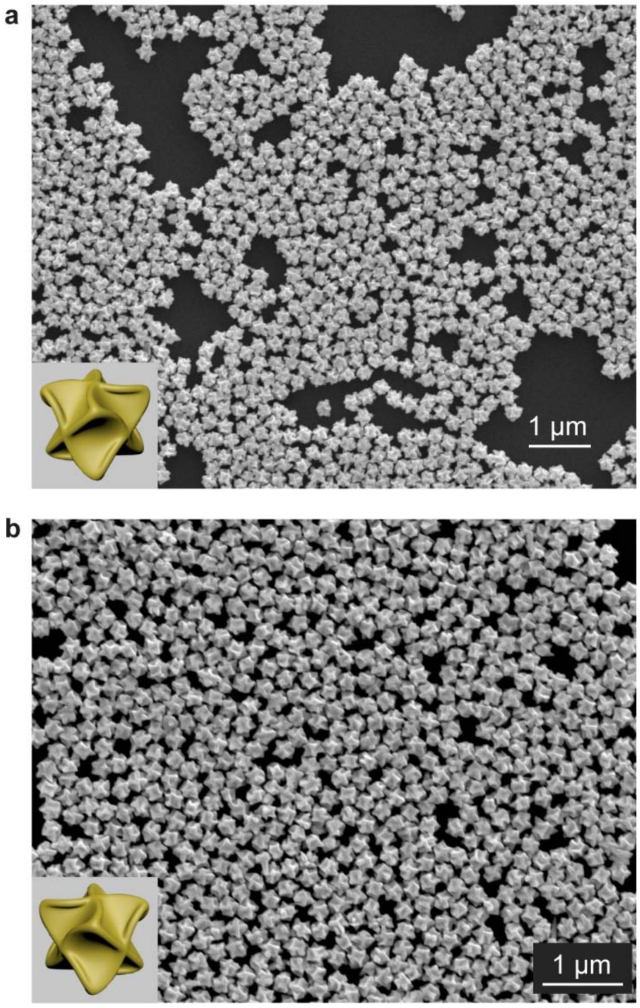

Supplementary Fig. 4 | Large-area SEM images of the 432 helicoid III nanocrystals. a, Synthesized from L-GSH. **b**, Synthesize from D-GSH.

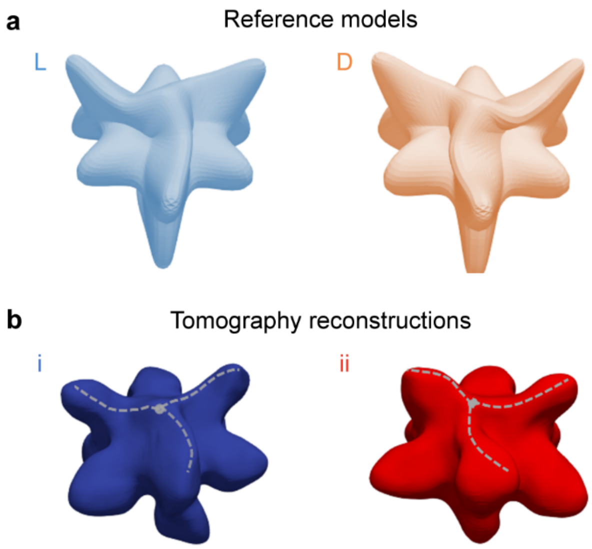

Supplementary Fig. 5 | Hausdorff chirality of the Au nanotriskelions. a, Idealised L- and D-reference models based on SEM imaging. **b**, Tomography reconstructions of the particles synthesized from L- and D-GSH (particle i and ii, respectively). The overlaid gray dashes follow the ridge of each branch as a visual aid. The normalized mean Hausdorff distances between particles i and reference L, particles ii and reference L, particles i and reference D, and particles ii and reference D are 0.021, 0.045, 0.029, and 0.039, respectively. The normalized mean Hausdorff distances confirm that the particles i and ii are L- and D-handed, respectively.

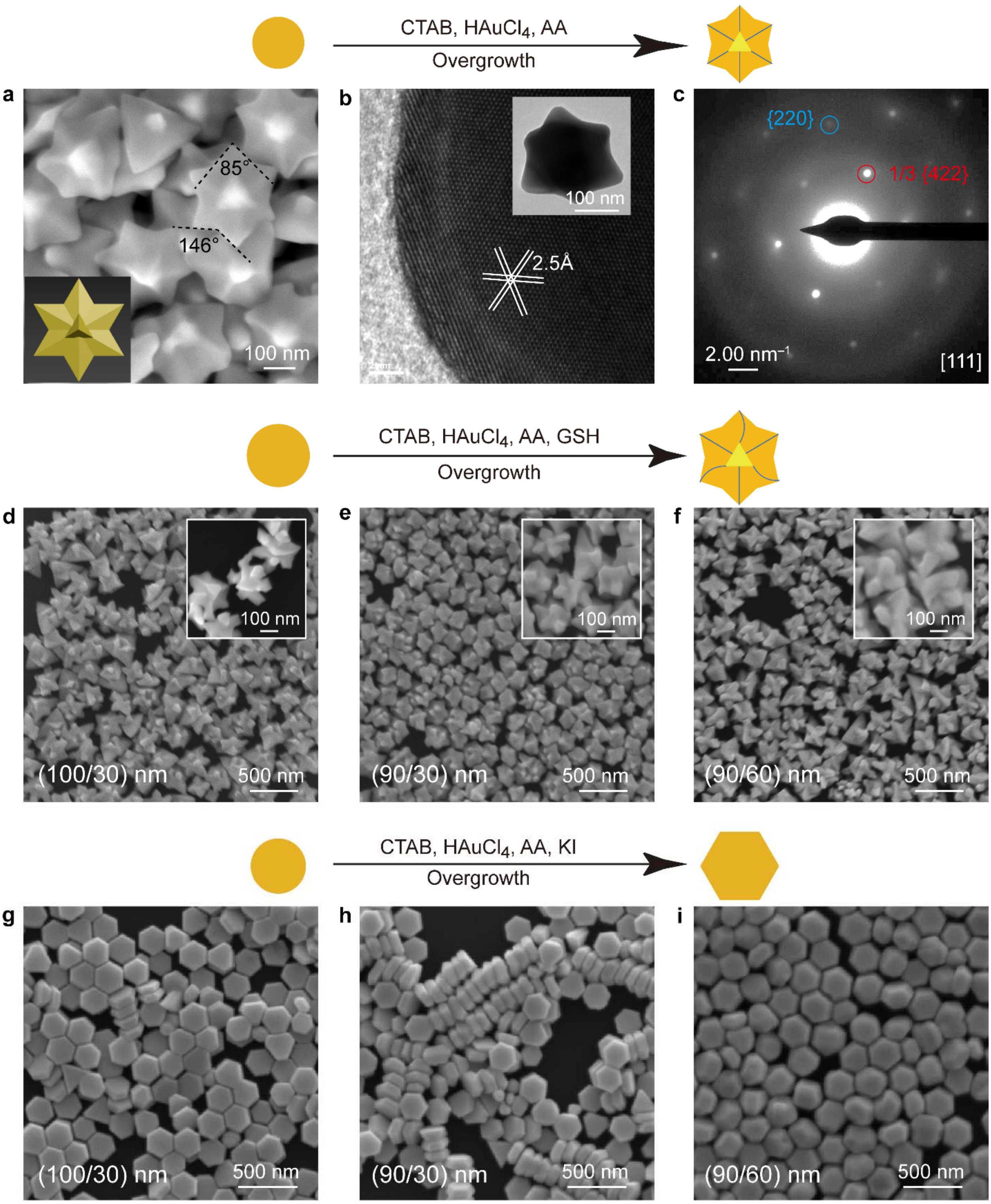

Supplementary Fig. 6 | Overgrowth on the Au nanodisks under different conditions. a–c, Overgrowth on the 100/30 nm Au nanodisks without the addition of GSH or KI. The resultant nanocrystals show a hexagram shape and high-Miller-index facets are generated from the {111} facets, as revealed by SEM imaging (**a**). The angle between the two salient edges of the Au hexagram-shaped crystals is ~85°, and the angle of the concave edges is ~146°. We hypothesized that the hexagram-shaped nanocrystals are enclosed by the {541} facets[1]. The HAADF-STEM images (**b**) and SAED pattern (**c**) recorded along the <111> directions reveal that the hexagram-shaped nanocrystals have stacking faults, resulting from

the nanodisk seeds[2]. The lattice spacing of 0.25 nm is triple that of the {422} lattice spacing of the Au nanocrystals, showing the appearance of the diffraction of the 1/3{422} planes that are normally forbidden by a face-centered cubic lattice. **d**–**f**, SEM images showing the overgrowth on the Au nanodisks in the presence of GSH. The differently sized nanodisks were used, including 100/30 (**d**), 90/30 (**e**), and 90/60 nm (**f**). GSH is the key to the tilting of the high-Miller-index edges. The nanodisks with large diameter-to-thickness ratios tend to evolve into Au nanotriskelions, while the thicker nanodisks of 90/60 nm preferentially develop into 432 helicoid nanocrystals. **g**–**i**, SEM images showing the overgrowth on the Au nanodisks in the presence of KI (2 μM). The Au nanodisks of 100/30 (**g**), 90/30 (**h**), and 90/60 nm (**i**) evolve into Au nanoplates with the help of the passivation by iodide ions on the {111} facets.

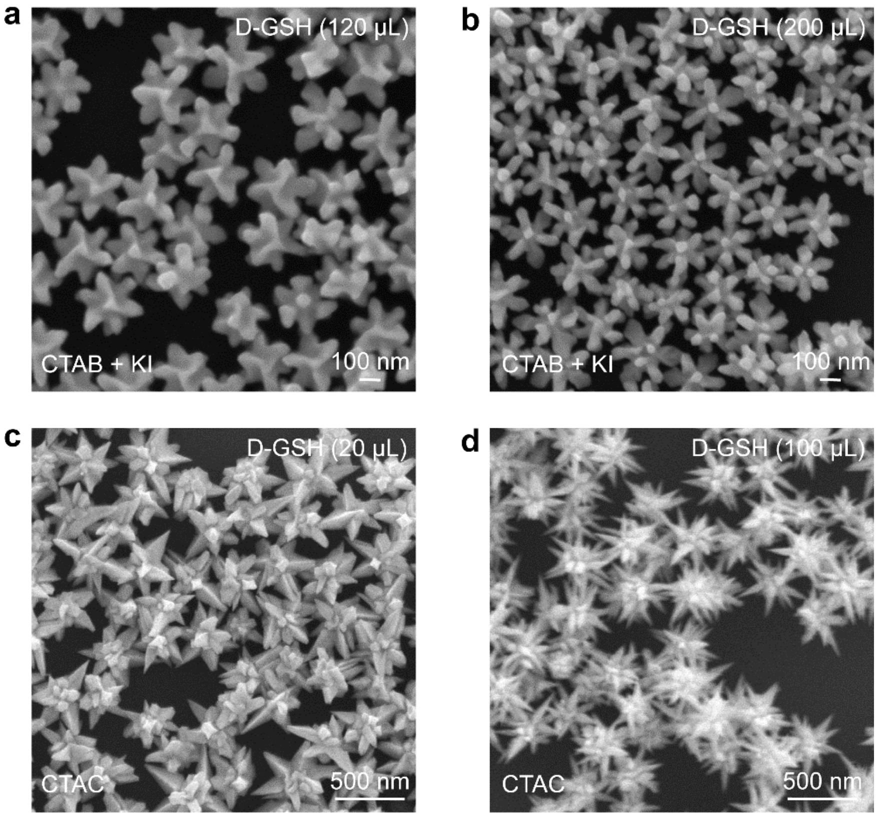

Supplementary Fig. 7 | Use of different halide ions to control the chiral growth of the Au nanodisks. a,**b**, SEM images of the nanoparticles grown from CTAB and KI. The growth solution with a total volume of 10 mL was made of the 90/30 nm nanodisks, CTAB (0.1 M, 0.8 mL), KI (1 mM, 40 µL), AA (0.1 M, 1 mL), $HAuCl_4$ (0.01 M, 0.4 mL), and D-GSH (2.75 mM). The generation of Au nanotriskelions in the presence of bromide and iodide ions was observed. With the increase in the concentration of D-GSH, the twisted morphology remains almost unchanged, and the protrusion gradually elongates. **c**,**d**, SEM images of the nanoparticles grown from CTAC. The growth solution with a total volume of 10 mL was made of the 90/30 nm nanodisks, CTAC (0.1 M, 0.8 mL), AA (0.1 M, 1 mL), $HAuCl_4$ (0.01 M, 0.4 mL), and D-GSH (2.75 mM). The dendritic morphology with various high-index facets was generated from the nanodisks (**c**). The dendrite structures with higher complexity were observed when a larger amount of GSH was used (**d**). We therefore conclude that chloride ions cannot be used to control the chiral growth of the Au nanodisks.

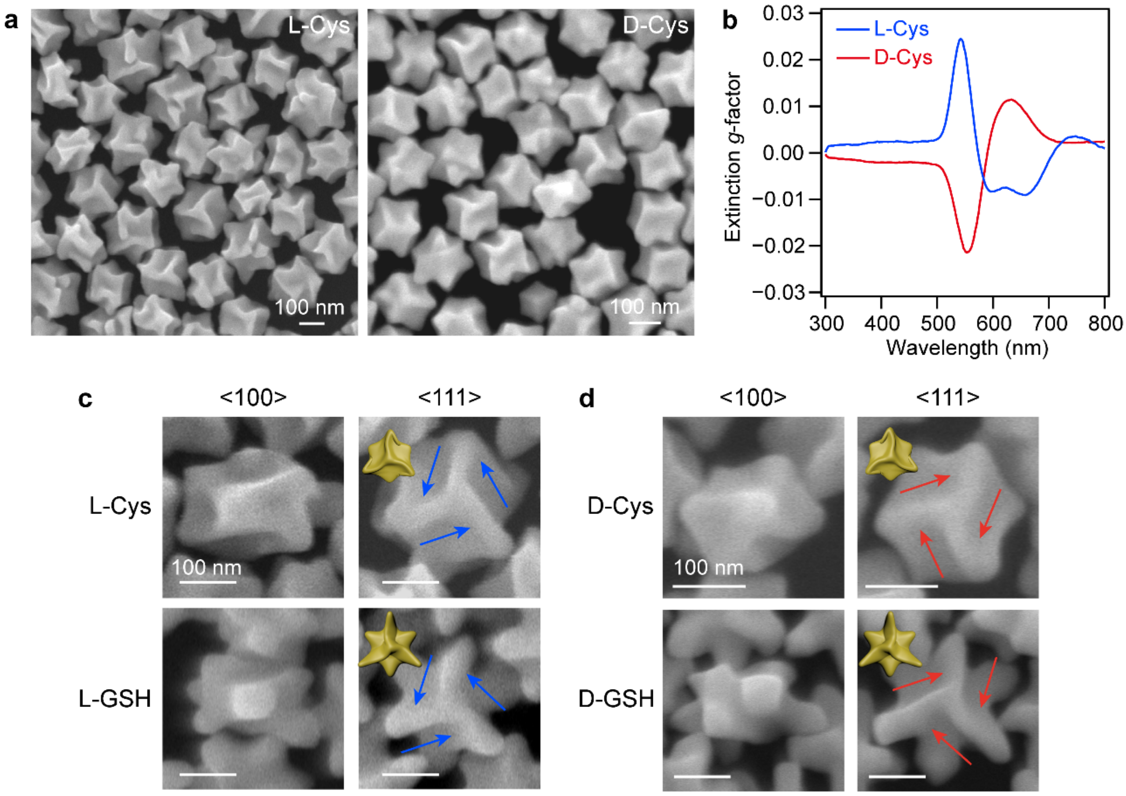

Supplementary Fig. 8 | Structural difference of the Au nanotriskelions synthesized in the presence of cysteine (Cys) and GSH. a,**b**, SEM images and *g*-factor spectra of the Au nanotriskelions obtained from L- and D-Cys. **c**,**d**, Structural difference of the Au nanotriskelions. The Cys- and GSH-based nanotriskelions show similar chiral structures with their twisted arms extending from the center of the {111} facet. Compared with Cys, GSH composed of three amino acids and a longer carbon chain can interact with more kink atoms on the Au nanodisks, resulting in the production of larger protrusions and longer arms. The arrows in the SEM images indicate the twisted edges on different chiral surfaces. All the scale bars for the SEM images in (**c**,**d**) are the same. Source data are provided as a Source Data file.

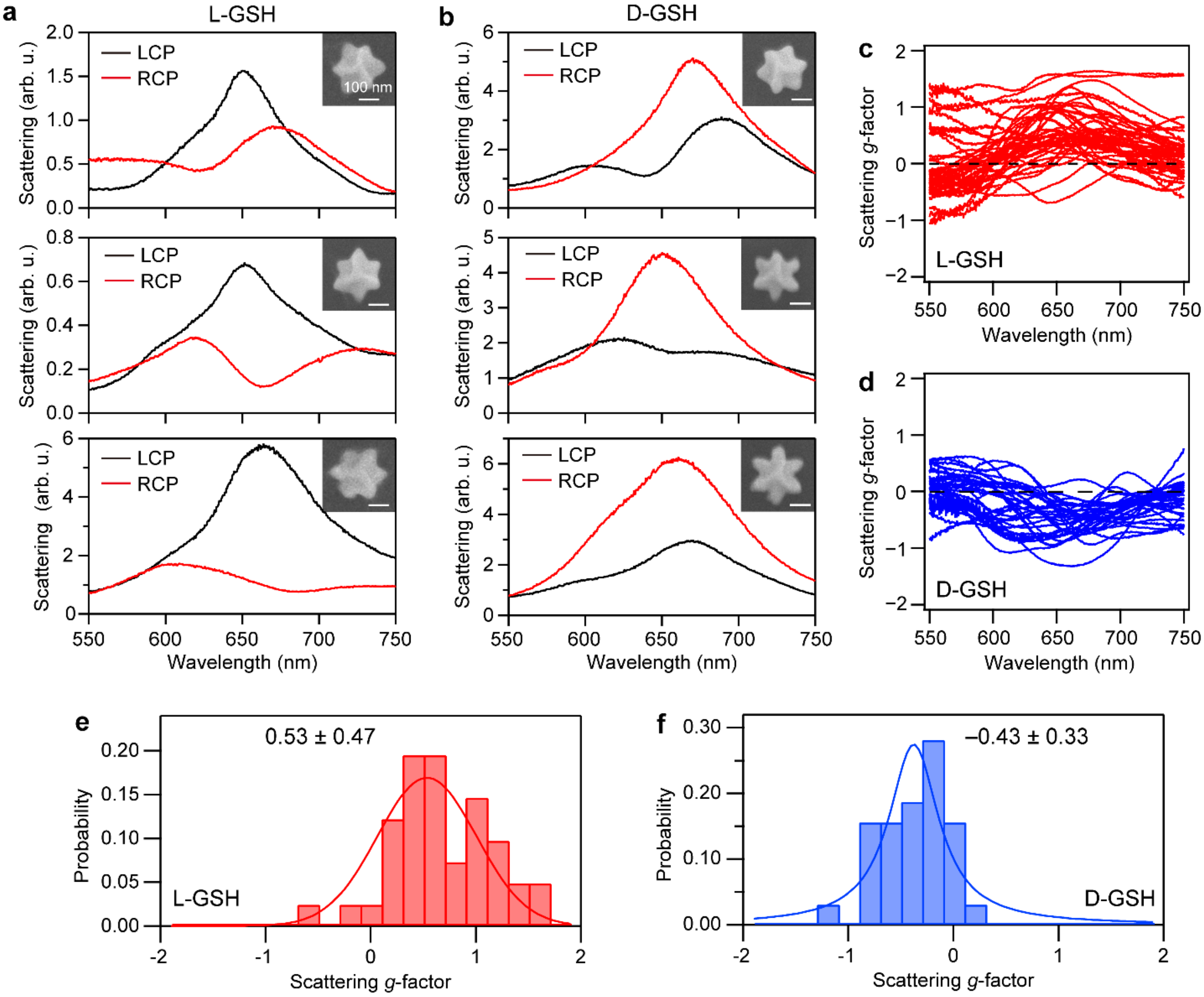

Supplementary Fig. 9 | CDS measurements of the Au nanotriskelions. a,b, SEM-correlated single-particle CDS spectra for the L- (**a**) and D-nanotriskelions (**b**) under LCP and RCP excitation at 550–750 nm. Inset: SEM images of the measured chiral nanocrystals. The scale bars in all the insets are the same. The L-nanotriskelions exhibit a strong scattering peak under LCP excitation and significantly reduced scattering under RCP excitation. The D-nanotriskelions present the opposite scattering responses. The SEM images of the measured nanotriskelions confirm that the differential scattering results from their different geometrical chiralities. **c**,**d**, Measured scattering dissymmetry factors from CDS characterization for a large number of nanotriskelions, including 41 L-type (**c**) and 32 D-type nanotriskelions (**d**). Most of the nanotriskelions were found to have strong chirally scattering responses at 600–700 nm. The absolute value of the scattering *g*-factor from most of the D- and L-nanotriskelions are larger than 0.5. **e**,**f**, Histograms for the L- and D- type nanotriskelions with the standard deviations for the scattering *g*-factor at 650 nm. Source data are provided as a Source Data file.

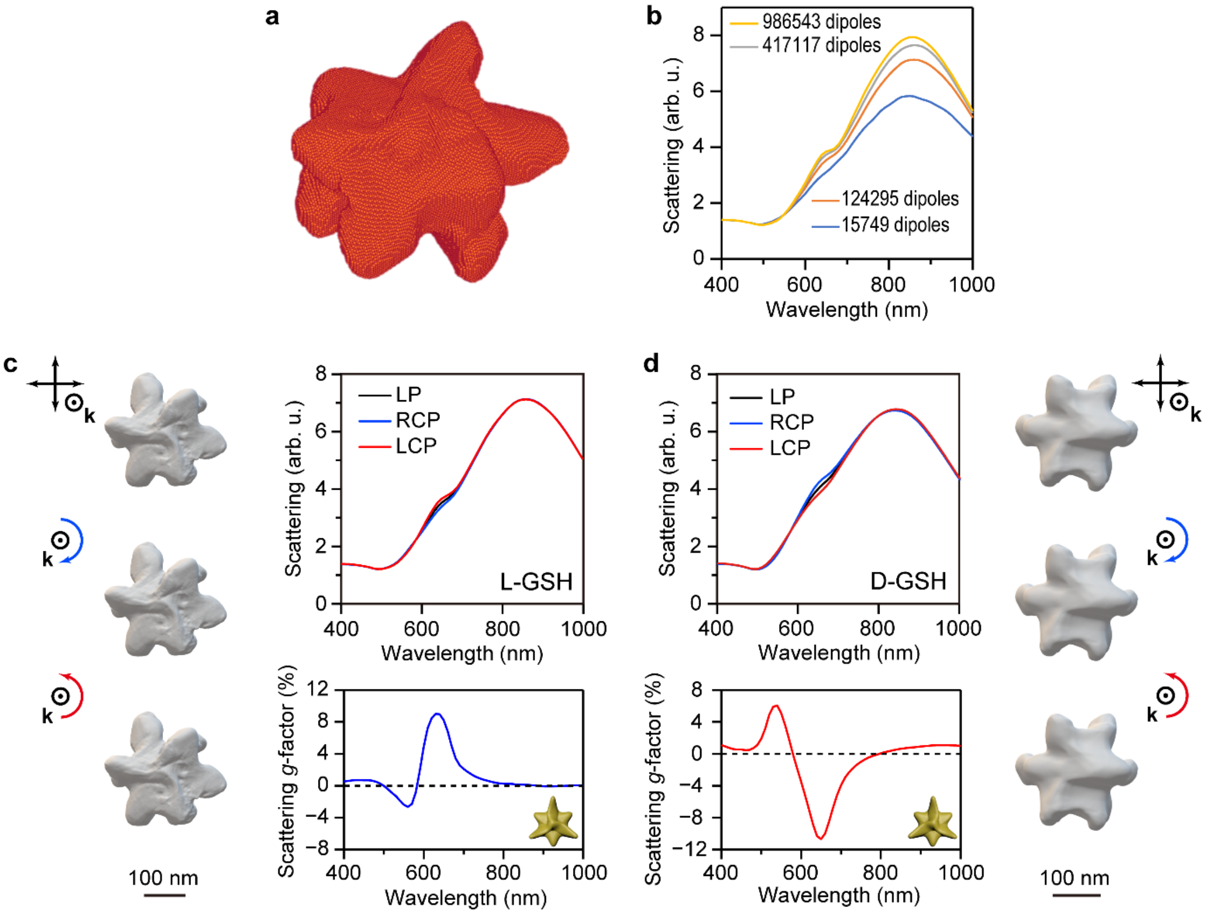

Supplementary Fig. 10 | Simulated chiroptical responses of the Au nanotriskelions of opposite handedness. a,**b**, The geometry reconstructed from TEM tomography were converted into a dipole array (**a**) for discrete dipole scattering simulation. The convergence tests were performed with different numbers of dipoles (**b**). About 400 000 dipoles were selected for the rest of the simulations. **c**,**d**, The scattering spectra of the Au nanotriskelion enantiomers were calculated under the excitation of linearly polarized (LP), left-handed circularly polarized (LCP), and right-handed circularly polarized (RCP) light. The corresponding scattering dissymmetry factor (*g*-factor) spectra of the L- (**c**) and D-nanotriskelions (**d**) were obtained. Source data are provided as a Source Data file.

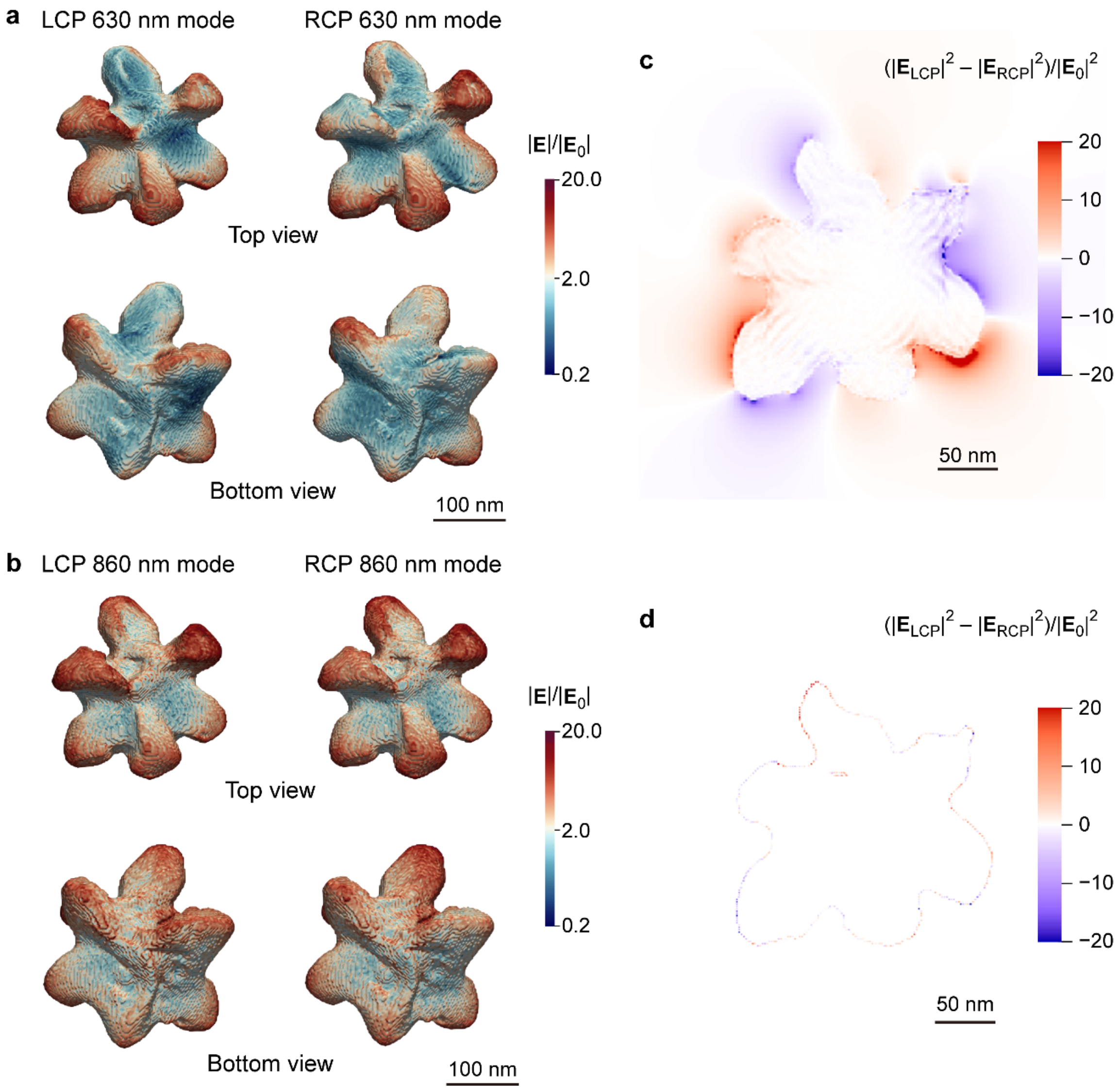

Supplementary Fig. 11 | Simulated near-field chiroptical properties of the Au L-nanotriskelions. The electric field around the L-nanotriskelion was calculated under the excitation of LCP and RCP light at 630 nm (**a**) and 830 nm (**b**). The distributions of the electric field enhancement are shown in the color maps at the logarithmic scale. The results were further used to calculate the difference in the field $(|\mathbf{E}_{\mathrm{LCP}}|^2 - |\mathbf{E}_{\mathrm{RCP}}|^2)/|\mathbf{E}_0|^2$ at 630 nm (**c**) and 830 nm (**d**). The asymmetric response in the electric field (**a**,**c**) shows that strong optical chirality is produced at the high-energy peak of 630 nm.

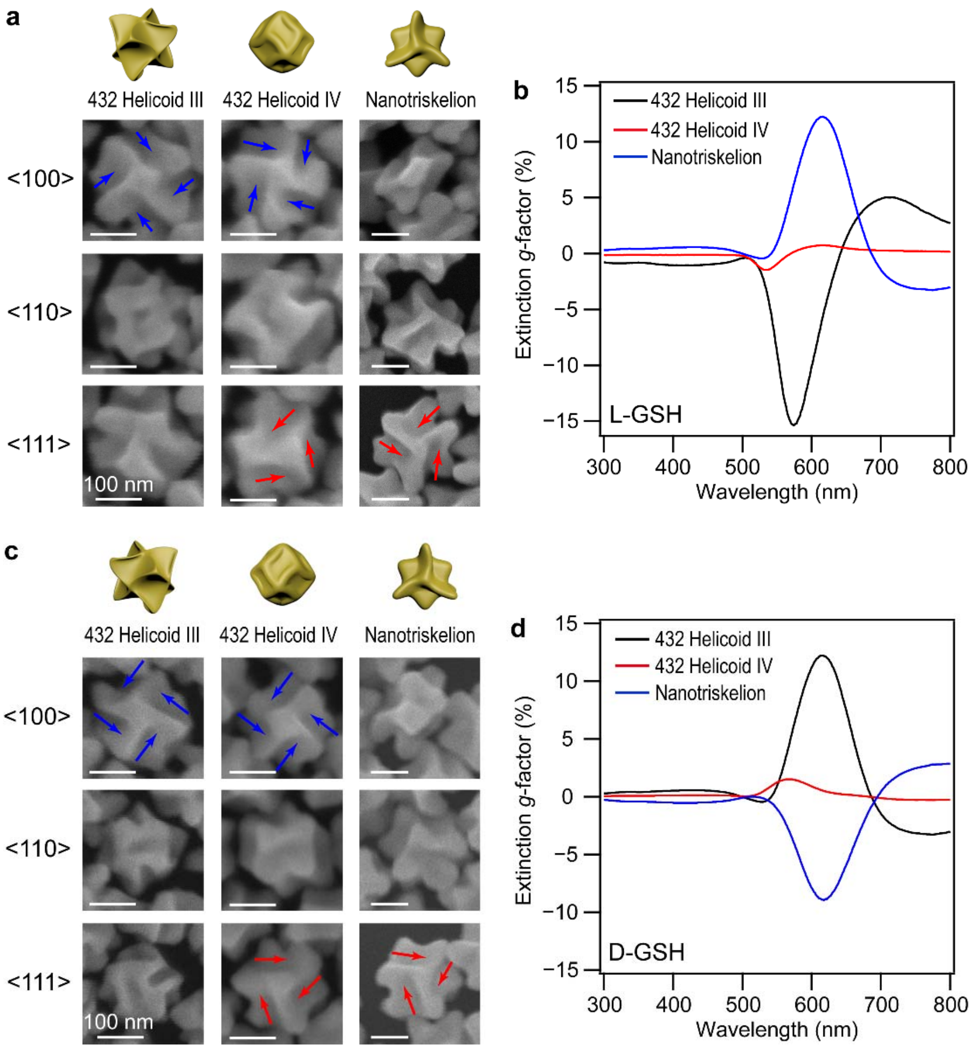

Supplementary Fig. 12 | Different chiral nanocrystals. **a**,**b**, SEM images and *g*-factor spectra of the nanocrystals synthesized in the presence of L-GSH. **c**,**d**, SEM images and *g*-factor spectra of the nanocrystals synthesized in the presence of D-GSH. The 432 helicoid III and IV nanocrystals were obtained by use of Au nanooctahedrons as the seeds. The Au nanotriskelions were grown from the Au nanodisks. The SEM images (**a**,**c**) show the chiral nanocrystals oriented along the <100>, <110>, and <111> directions. The arrows in the SEM images indicate the twisted edges at different chiral surfaces. The different chiroptical responses of the nanocrystals thus arise from their different chiral geometries. In the L-type 432 helicoid III nanocrystals, the fourfold edge rotating clockwise around the twisted center is observed along the <100> directions, while the threefold edge rotating counterclockwise is observed along the <111> directions in the L-nanotriskelions. The 432 helicoid III nanocrystals and nanotriskelions prepared in the presence of the same L-GSH therefore present the opposite chiroptical properties. In contrast, the 432 helicoid IV nanocrystals show two surfaces with opposite chirality along the <111> and <100> directions, resulting in a smaller *g*-factor. All the scale bars in (**a**,**c**) are the same. Source data are provided as a Source Data file.

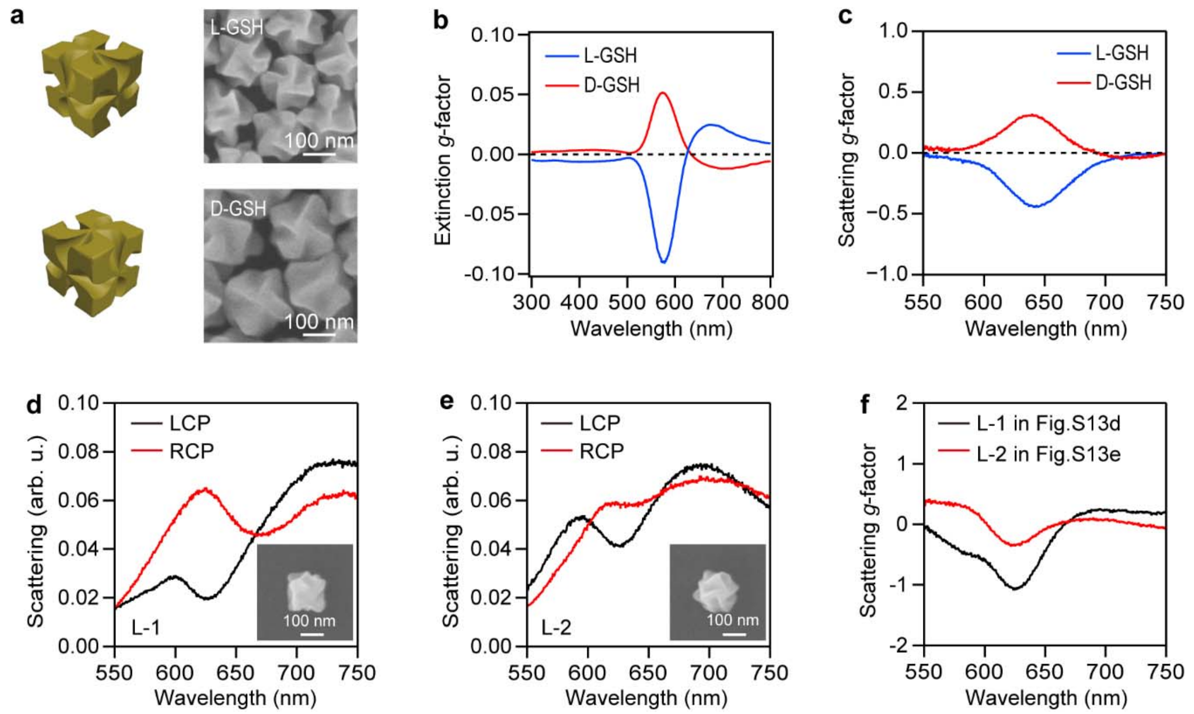

Supplementary Fig. 13 | CDS measurements of the 432 helicoid III NPs. a, Constructed models and SEM images of the 432 helicoid III NPs. **b**, Dissymmetry factor spectra measured from CD spectrometry for the 432 helicoid III NPs in solution. **c**, Average scattering *g*-factor spectra obtained from the CDS measurements on the 432 helicoid III NPs, including 14 L-type and 11 D-type NPs. **d**,**e**, SEM-correlated single-particle CDS spectra for the L-432 helicoid III with different tilted angles under LCP and RCP excitation at 550–750 nm. Insets: SEM images of the measured chiral nanocrystals. The SEM images of the measured L-432 helicoid III confirm that the differential scattering results from their different tilted angles. **f**, Scattering *g*-factor spectra for the L-432 helicoid III in (**d**,**e**). When the chiral surface faces upwards, the L-432 helicoid III shows the stronger differential scattering response. Source data are provided as a Source Data file.

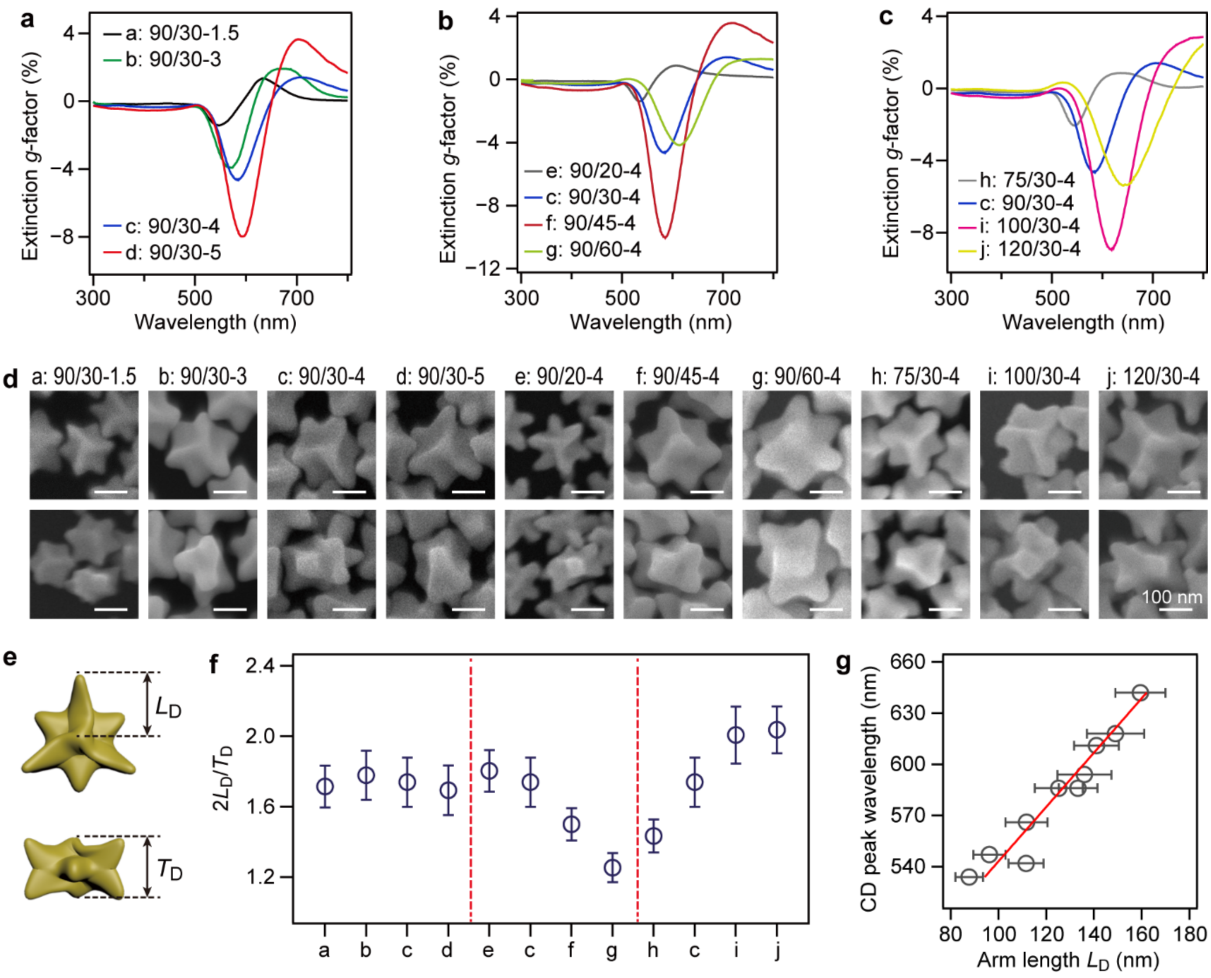

Supplementary Fig. 14 | Controllable synthesis of the Au D-nanotriskelions. a–c, Dissymmetry factor spectra for the D-nanotriskelions with various dimensions, which were synthesized by use of the Au precursor with different amounts (**a**) and the Au nanodisk seeds with different thicknesses (**b**) and diameters (**c**). The nanotriskelions marked by, e.g., 90/20-4, means that the seeds are the Au nanodisks with an average diameter of 90 nm and an average thickness of 20 nm and the added amount of the Au precursor is 4 μmol for the Au nanodisk seeds with an optical density of 0.8. **d**, SEM images of the D-nanotriskelions with various dimensions, including the observation from the top (top) and side (bottom). All the scale bars in (**d**) are the same. The large-scale SEM images are shown in Supplementary Fig. 15. **e**, Schematics of the Au nanotriskelions with the arm length L_D and the thickness T_D indicated. **f**, Aspect ratios of the Au nanotriskelions, defined as $2L_D/T_D$ in (**d**). All error bars show mean ± standard deviation. $n = 100$ independent experiments. **g**, Dependence of the peak wavelength in the CD spectra on the nanotriskelion arm length. The trend is indicated with the red solid lines. All error bars show mean ± standard deviation. $n = 100$ independent experiments. Source data are provided as a Source Data file.

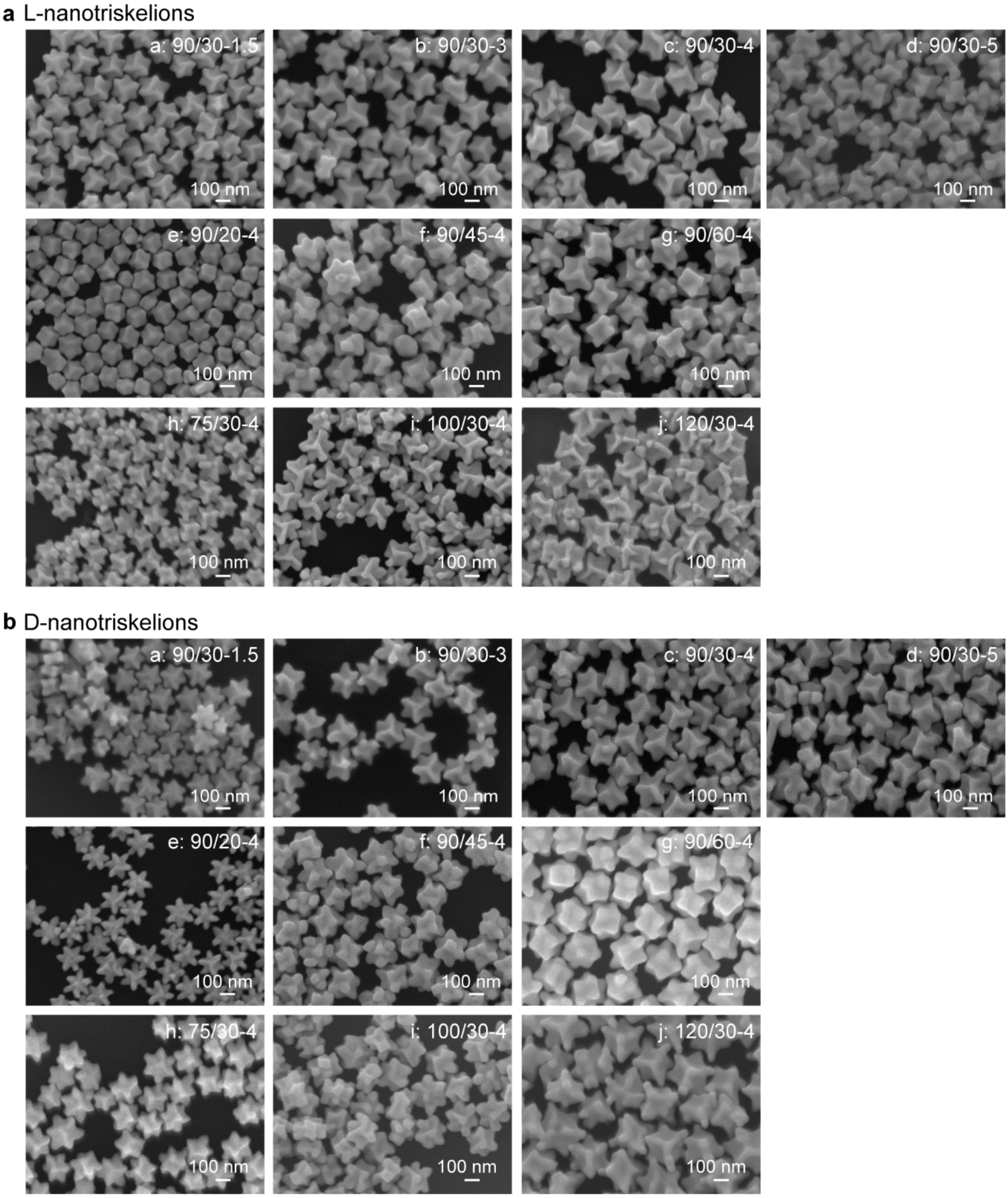

Supplementary Fig. 15 | Large-area SEM images. **a**, L-nanotriskelions. **b**, D-nanotriskelions. They have different dimensions and have been shown in Fig. 3 and Supplementary Fig. 14. The nanotriskelions were grown from differently sized Au nanodisks.

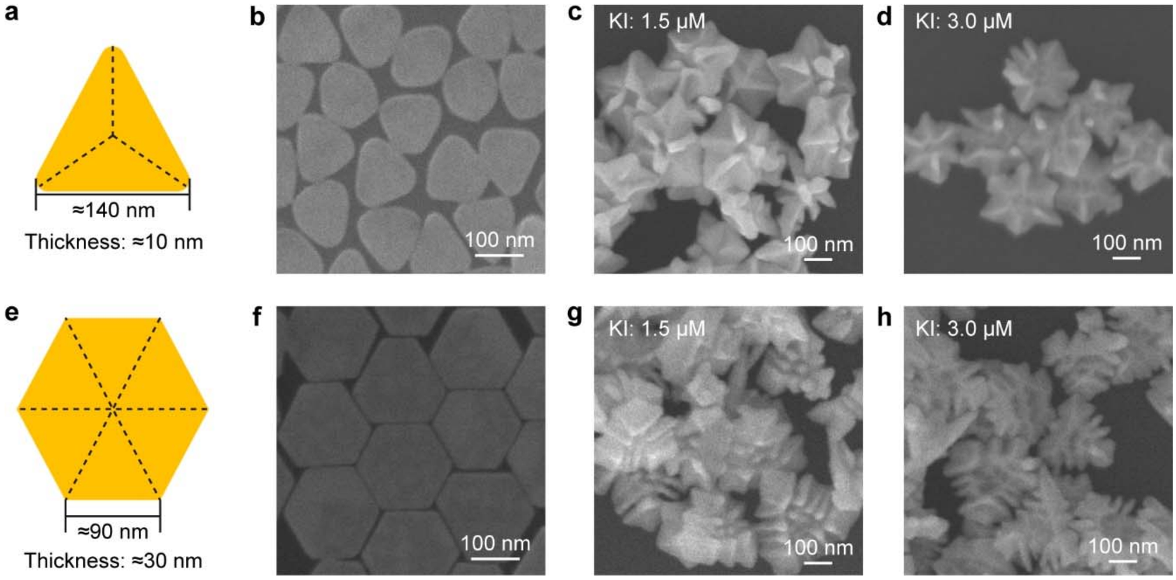

Supplementary Fig. 16 | Morphology evolution of the chiral nanocrystals grown from other nanoplates by HADG. a,**b**, Schematic and SEM image of Au triangular nanoplates with side lengths of 140 nm and thicknesses of 10 nm. **c**,**d**, SEM images showing the morphology evolution of the chiral nanoparticles grown from the triangular nanoplates. The growth solution with a total volume of 10 mL was made of CTAB (0.1 M, 0.8 mL), KI, AA (0.1 M, 1 mL), $HAuCl_4$ (0.01 M, 0.4 mL), L-GSH (2.75 mM, 150 μL), and DI water. The Au triangular nanoplates can evolve into Au nanotriskelions with segmented arms (**c**). The increased KI concentration can cause the gradual embedment of the twisted arms into the nanodisk domain (**d**). **e**,**f**, Schematic and SEM image of Au hexagonal nanoplates with side lengths of 90 nm and thicknesses of 30 nm. **g**,**h**, SEM images showing the morphology evolution of the chiral nanoparticles grown from the hexagonal nanoplates. The growth solution with a total volume of 10 mL was made of CTAB (0.1 M, 0.8 mL), KI, AA (0.1 M, 1 mL), $HAuCl_4$ (0.01 M, 0.4 mL), L-GSH (2.75 mM, 150 μL), and DI water. The Au hexagonal nanoplates can evolve into the nanoparticles with a dendritic morphology. We found that the Au triangular nanoplates and hexagonal nanoplates cannot be used to synthesize Au nanotriskelions with an ideal morphology, which is believed to be caused by their large aspect ratios. We finally employed the Au nanodisks as the seeds.

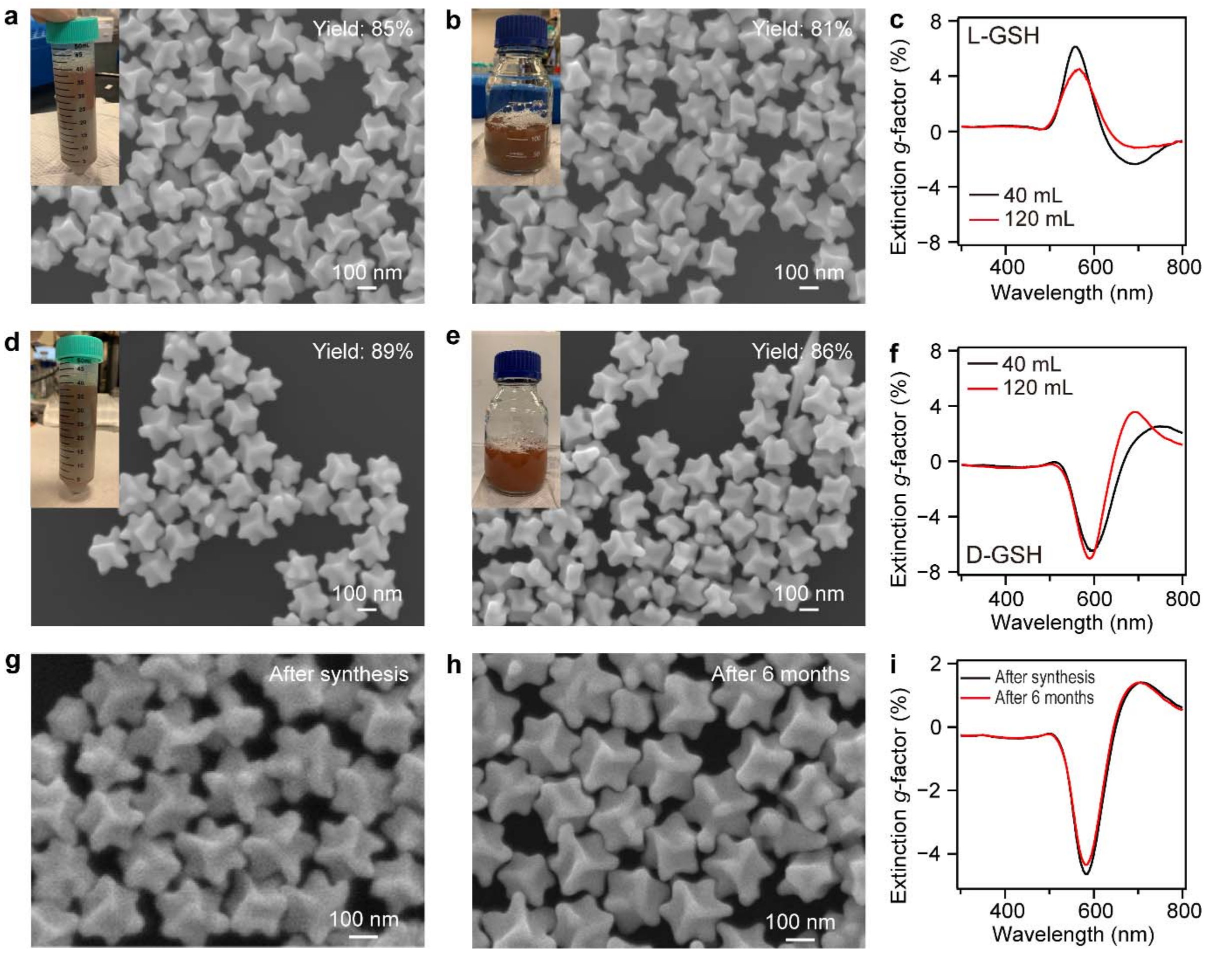

Supplementary Fig. 17 | Scale-up synthesis and structural stability of the Au nanotriskelions. a–**c**, SEM images and extinction *g*-factor spectra of the L-nanotriskelions. **d**–**f**, SEM images and extinction *g*-factor spectra of the D-nanotriskelions. The insets in (**a**,**b**,**d**,**e**) are the photographs of the nanotriskelion solutions. The HADG synthetic strategy was demonstrated to support high-quality and mass production. The synthesis of the Au nanotriskelions has been scaled up, with the total volume of the growth solutions expanded to 40 mL and 120 mL, respectively. The high quality was also ensured, as demonstrated by the SEM images and extinction *g*-factor spectra. We counted the number of the Au nanotriskelions with the desired structures from the SEM images. The number yield of the Au nanotriskelions was estimated to be larger than 80%. **g**–**i**, SEM images and extinction *g*-factor spectra of the D-nanotriskelions and the same sample stored after 6 months. The nanotriskelions were stored in an environment of 0–5 °C. The morphology (**g**,**h**) and chiroptical response (**i**) can be preserved well for at least 6 months. Source data are provided as a Source Data file.

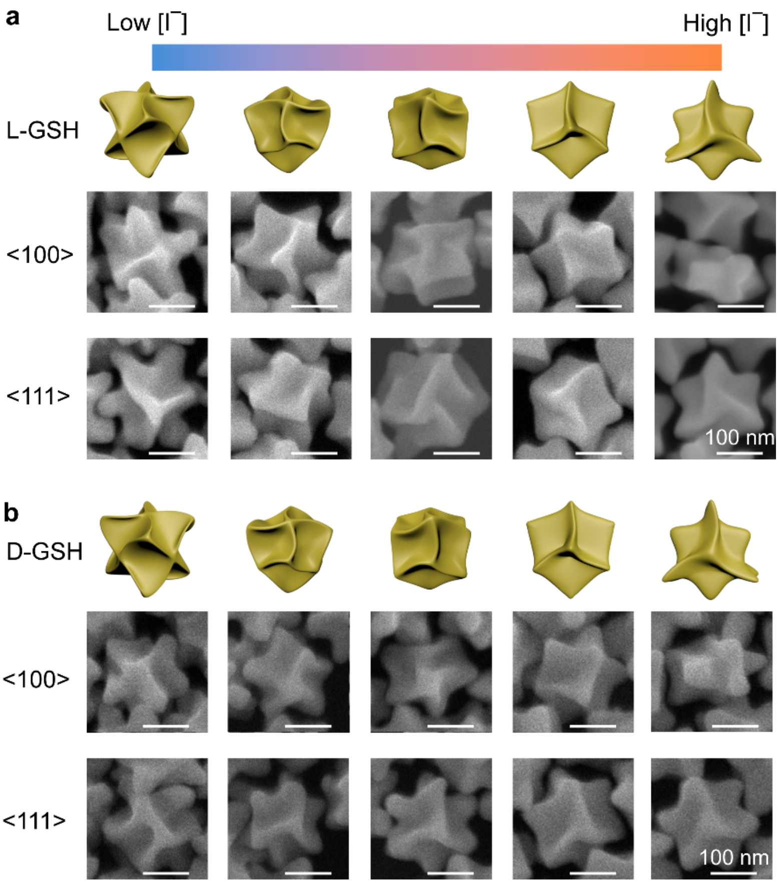

Supplementary Fig. 18 | Different chiral nanocrystals. a, Synthesized in the presence of L-GSH. **b**, Synthesized in the presence of D-GSH. The SEM images show the chiral nanocrystals in Fig. 4a with the orientation along the <100> and <111> directions. The 90/30 nm Au nanodisks were found to successively evolve into 432 helicoid III, 432 helicoid IV, and nanotriskelions with increasing KI concentrations. We can see that the dominant symmetry changes from fourfold rotational (FFR) to TFR (threefold rotational) symmetry with increasing KI concentrations. The scale bars in all the SEM images are the same.

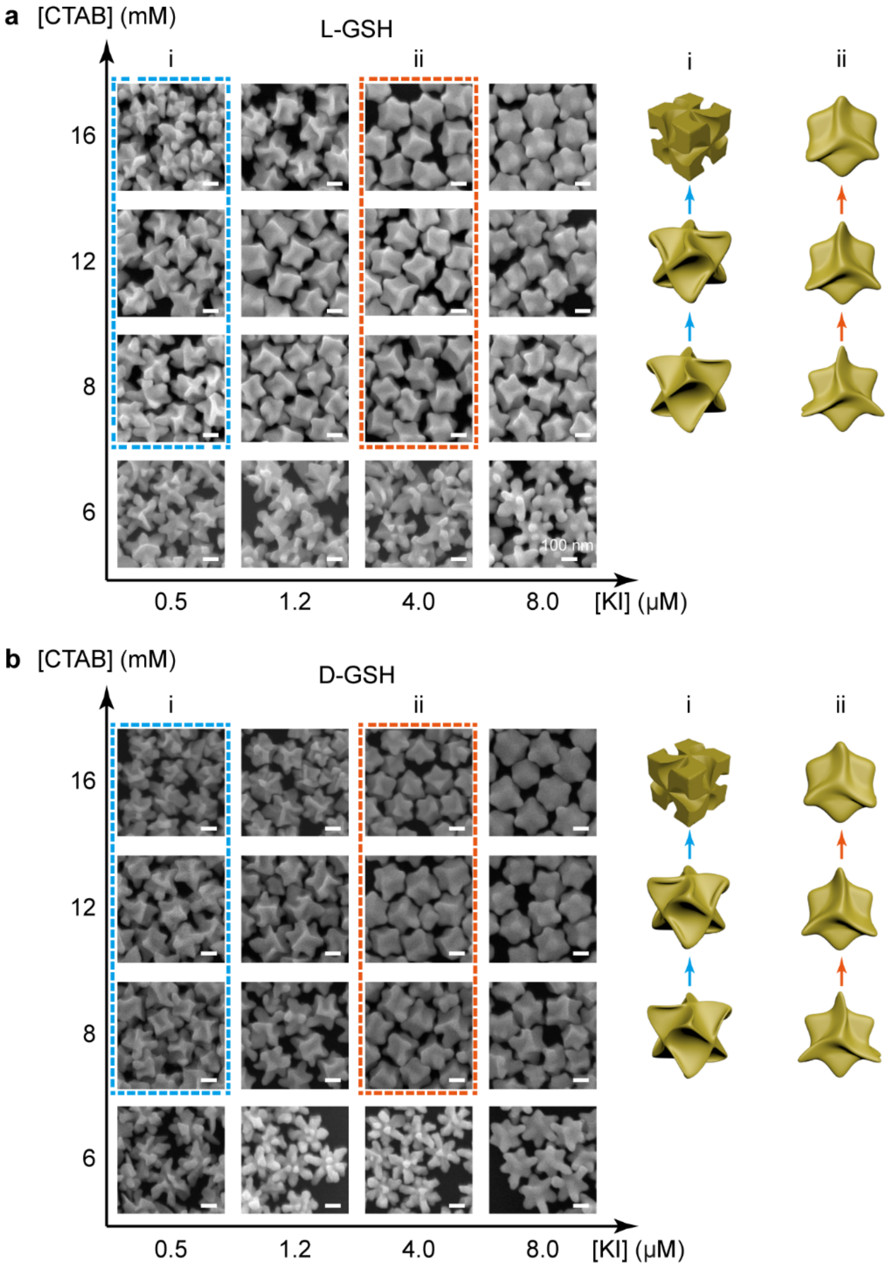

Supplementary Fig. 19 | Effect of increasing CTAB concentrations on the morphology evolution of the 432 helicoid III nanocrystals and nanotriskelions through HADG with the 90/30 nm Au nanodisks. a, SEM images and schematics for the nanocrystals synthesized with L-GSH. **b**, SEM images and schematics for the nanocrystals synthesized with D-GSH. All the scale bars are the same.

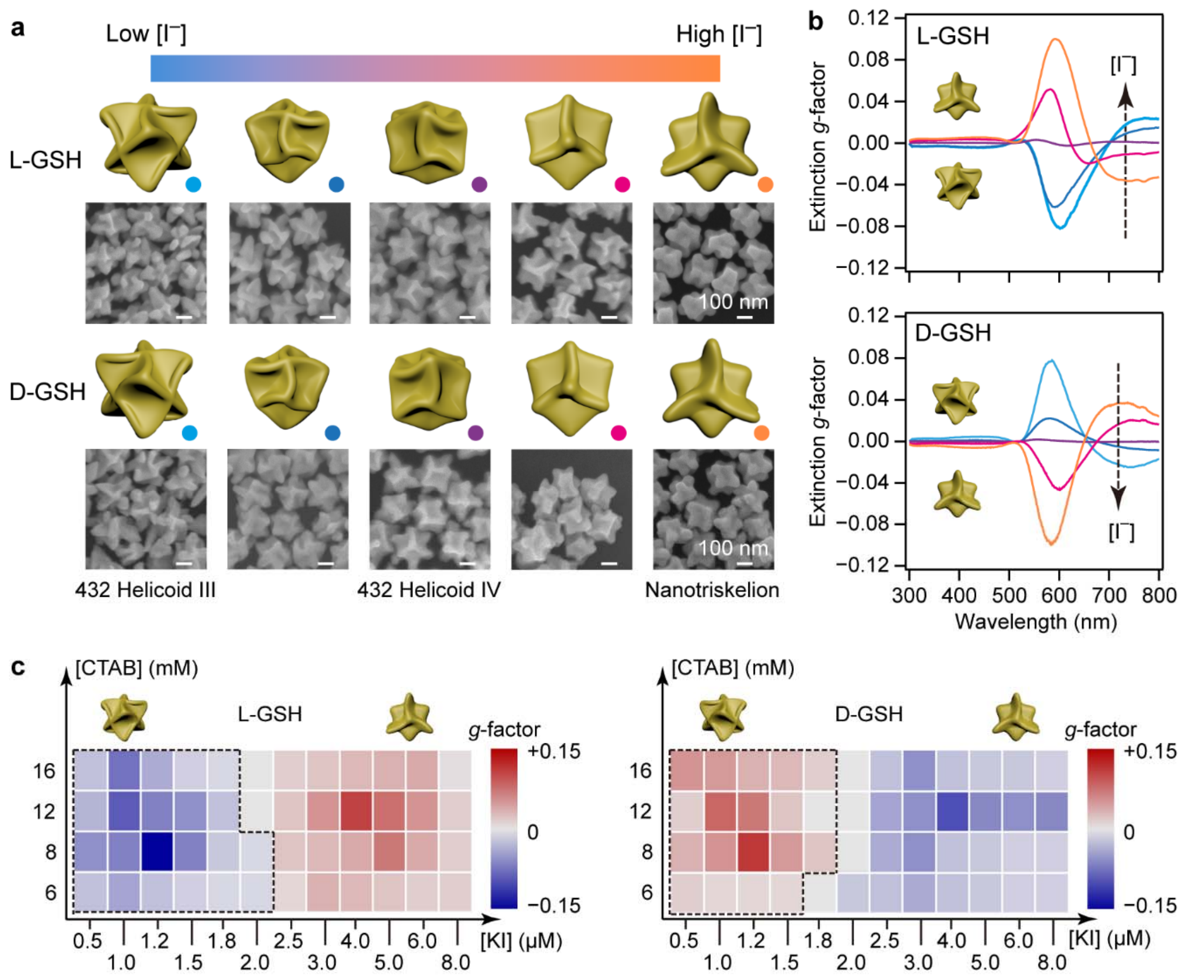

Supplementary Fig. 20 | Morphology and chirality evolution of the chiral nanocrystals grown from the Au nanodisks by HADG. The Au nanodisks with a size of 90/45 nm and diameter-to-thickness ratio of 2.0 were employed as the seeds. **a**, Effect of the KI concentration on the morphology of the obtained chiral nanocrystals. The 432 helicoid III nanocrystals are formed at low I^- concentrations and the 432 helicoid IV nanocrystals start to appear as the I^- concentration is increased. Further addition of KI finally results in the generation of Au nanotriskelions. The morphology evolution was confirmed by SEM imaging. All the scale bars in (**a**) are the same. **b**, Spectral evolution of the extinction *g*-factors for the chiral nanocrystals in (**a**). The chirality inversion of the optical response demonstrates that the grown chiral nanocrystals change from the 432 helicoid III nanocrystals to the Au nanotriskelions. The dashed lines show the spectral evolution with increasing KI concentrations. **c**, Dependence of the extinction *g*-factors of the grown chiral nanocrystals on the concentrations of CTAB and KI. The dashed frames show the grown chiral nanocrystals with FFR symmetry. Source data are provided as a Source Data file.

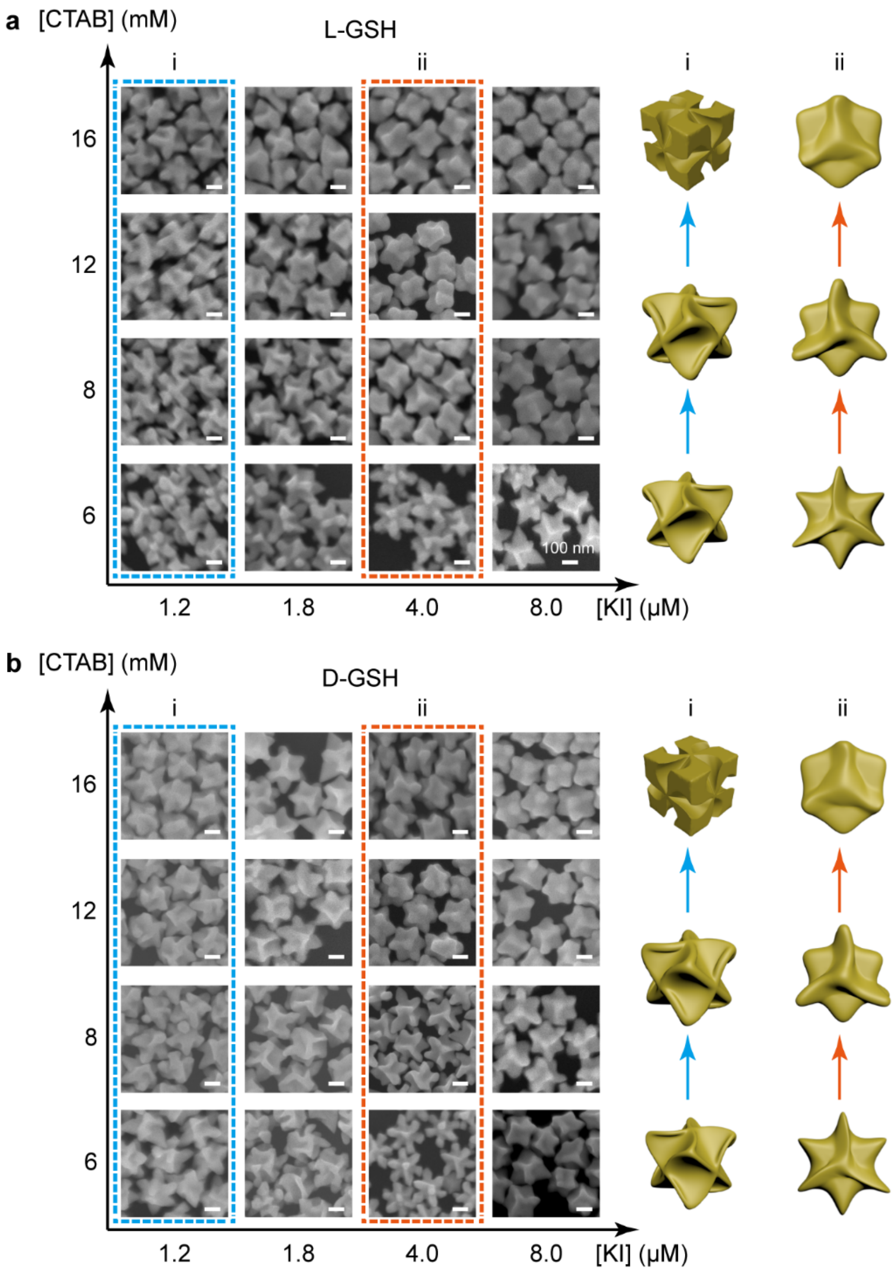

Supplementary Fig. 21 | Effect of increasing CTAB concentrations on the morphology evolution of the 432 helicoid III nanocrystals and nanotriskelions through HADG with the 90/45 nm Au nanodisks. The 432 helicoid III nanocrystals become more and more cubic as the CTAB concentration is increased. **a**, SEM images and schematics for the nanocrystals synthesized with L-GSH. **b**, SEM images and schematics for the nanocrystals synthesized with D-GSH. All the scale bars are the same.

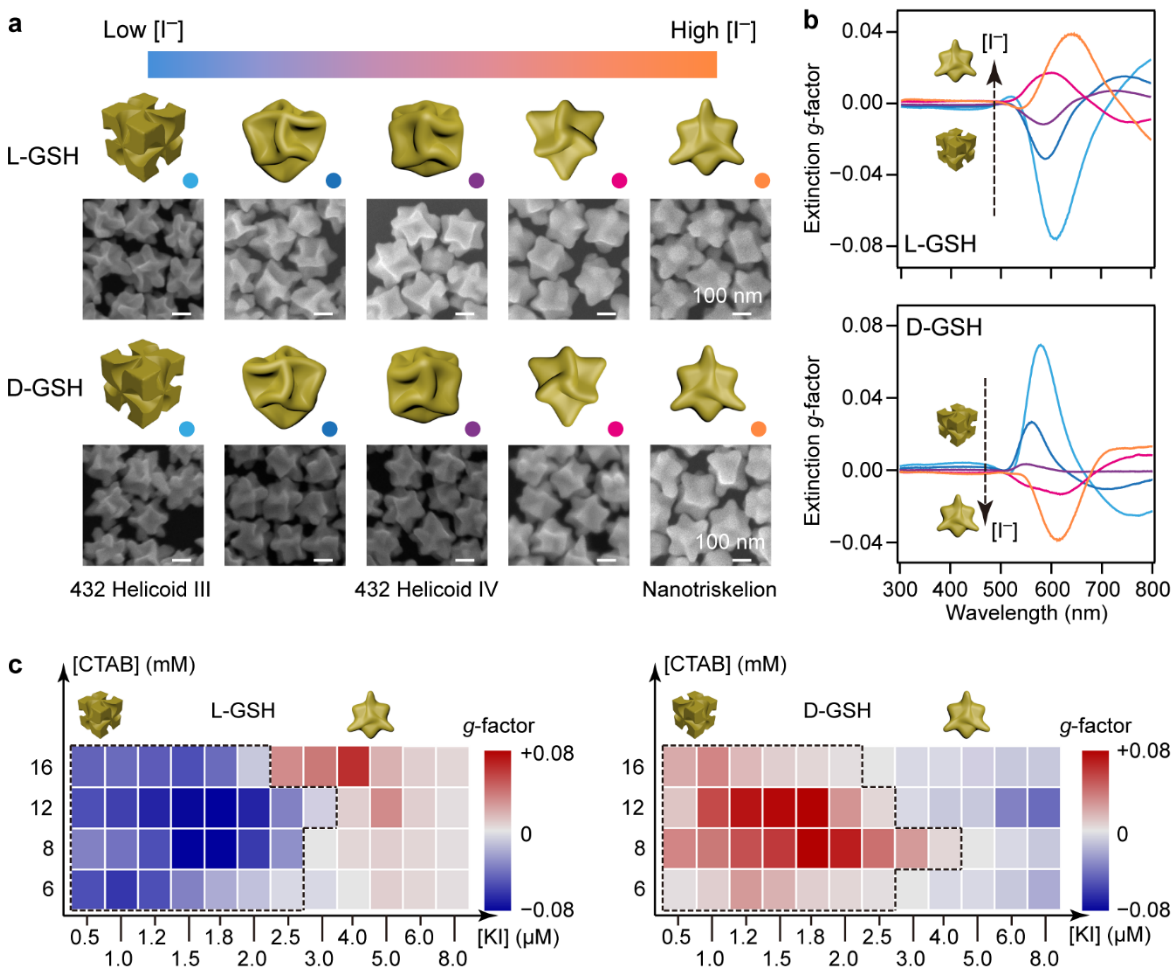

Supplementary Fig. 22 | Morphology and chirality evolution of the chiral nanocrystals grown from the Au nanodisks by HADG. The Au nanodisks with a size of 90/60 nm and diameter-to-thickness ratio of 1.5 were employed as the seeds. **a**, Effect of the KI concentration on the morphology of the obtained chiral nanocrystals. The 432 helicoid III nanocrystals are formed at low I^- concentrations and the 432 helicoid IV nanocrystals start to appear as the I^- concentration is increased. Further addition of KI finally results in the generation of Au nanotriskelions. The morphology evolution was confirmed by SEM imaging. All the scale bars in (**a**) are the same. **b**, Spectral evolution of the extinction *g*-factors for the chiral nanocrystals in (**a**). The chirality inversion of the optical response demonstrates that the grown chiral nanocrystals change from the 432 helicoid III nanocrystals to the Au nanotriskelions. The dashed lines show the spectral evolution with increasing KI concentrations. **c**, Dependence of the extinction *g*-factors of the grown chiral nanocrystals on the concentrations of CTAB and KI. The dashed frames show the grown chiral nanocrystals with FFR symmetry. Source data are provided as a Source Data file.

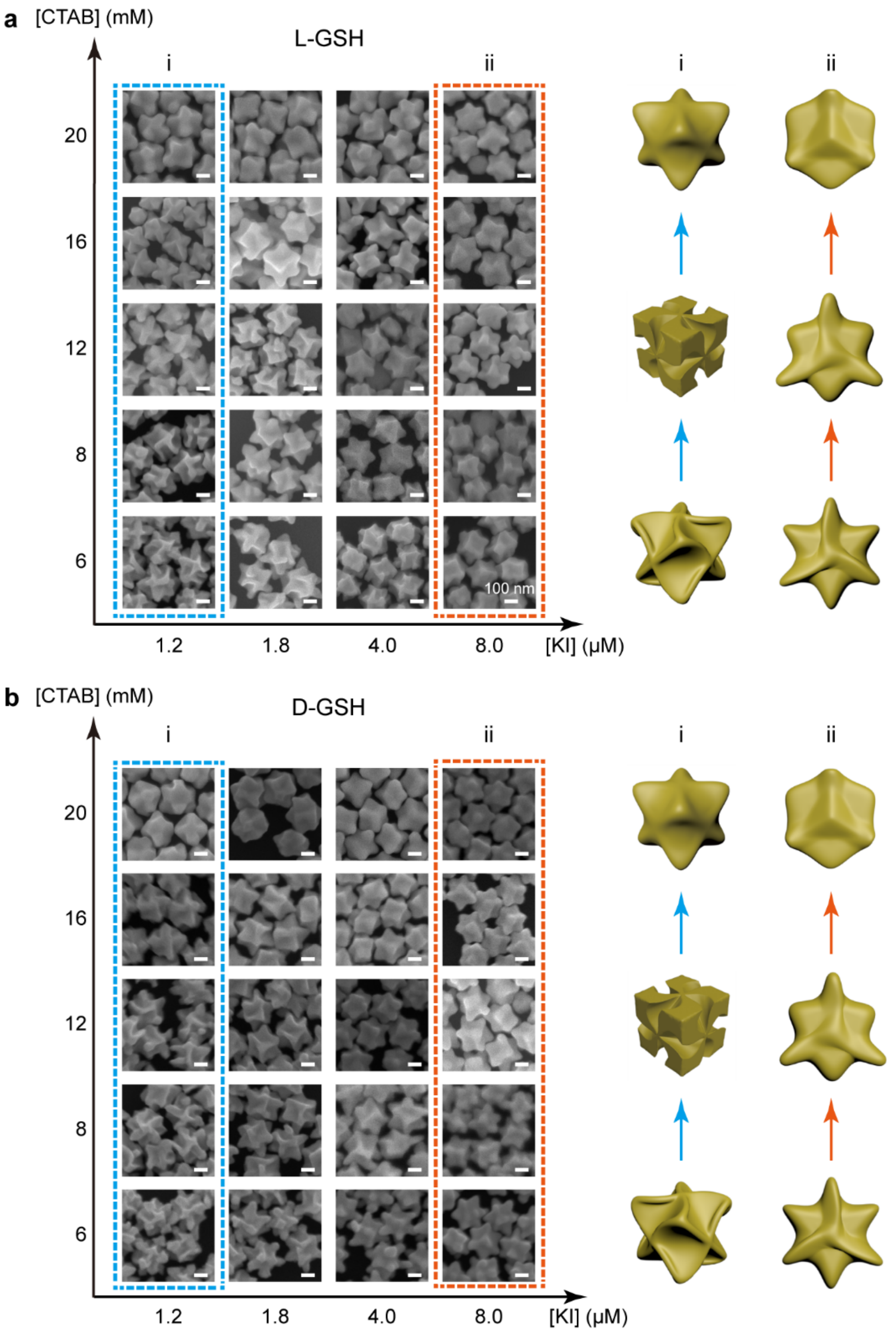

Supplementary Fig. 23 | Effect of increasing CTAB concentrations on the morphology evolution of the 432 helicoid III nanocrystals and nanotriskelions through HADG with the 90/60 nm Au nanodisks. The 432 helicoid III nanocrystals become more and more cubic as the CTAB concentration is increased. **a**, SEM images and schematics for the nanocrystals synthesized with L-GSH. **b**, SEM images and schematics for the nanocrystals synthesized with D-GSH. All the scale bars are the same.

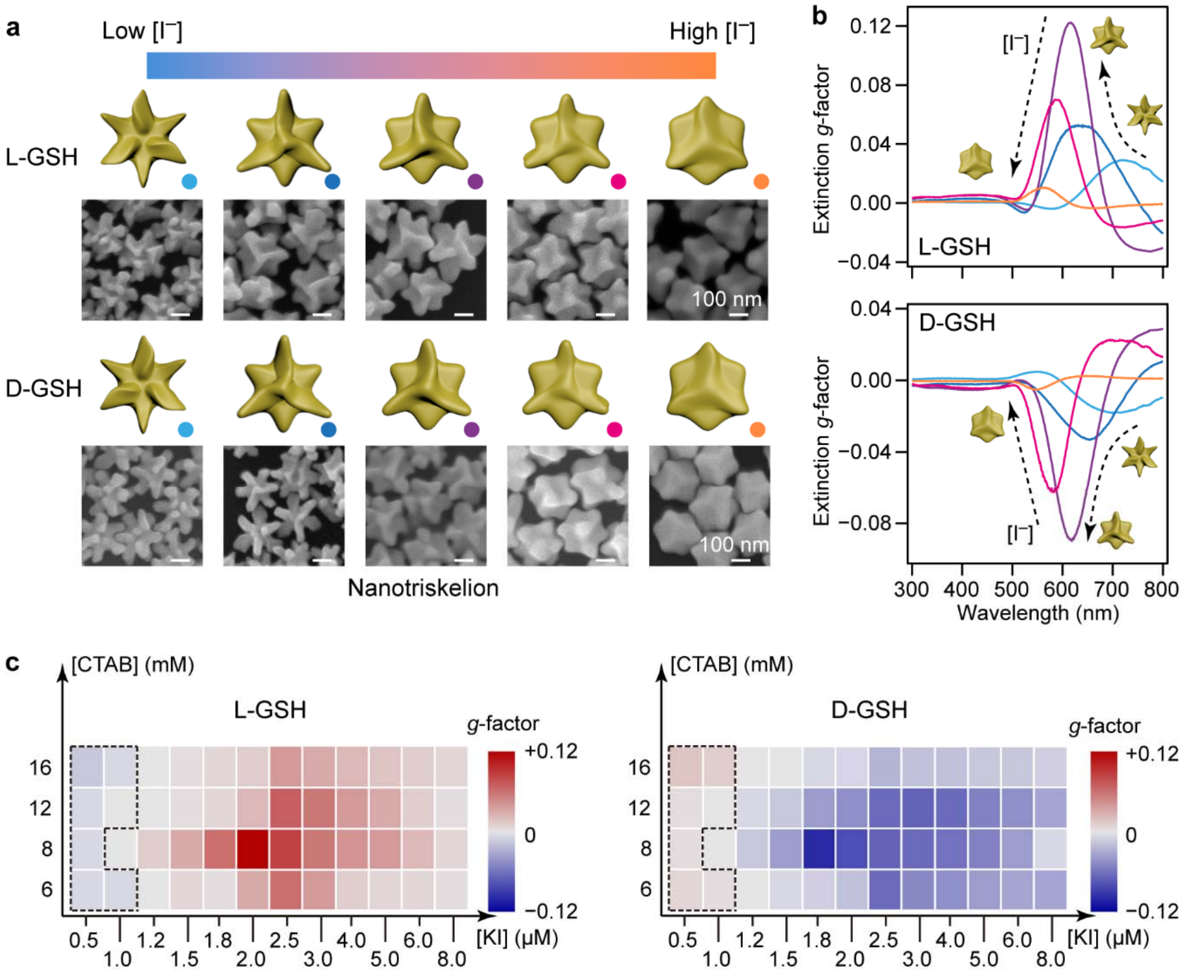

Supplementary Fig. 24 | Morphology and chirality evolution of the chiral nanocrystals grown from the Au nanodisks by HADG. The Au nanodisks with a size of 100/30 nm and diameter-to-thickness ratio of 3.3 were employed as the seeds. **a**, Effect of the KI concentration on the morphology of the obtained chiral nanocrystals. All the scale bars in the SEM images in (**a**) are the same. **b**, Spectral evolution of the extinction *g*-factors for the chiral nanocrystals in (**a**). The dashed lines show the spectral evolution with increasing KI concentrations. **c**, Dependence of the extinction *g*-factors of the grown chiral nanocrystals on the concentrations of CTAB and KI. The dashed frames show the grown chiral nanocrystals with FFR symmetry. Source data are provided as a Source Data file.

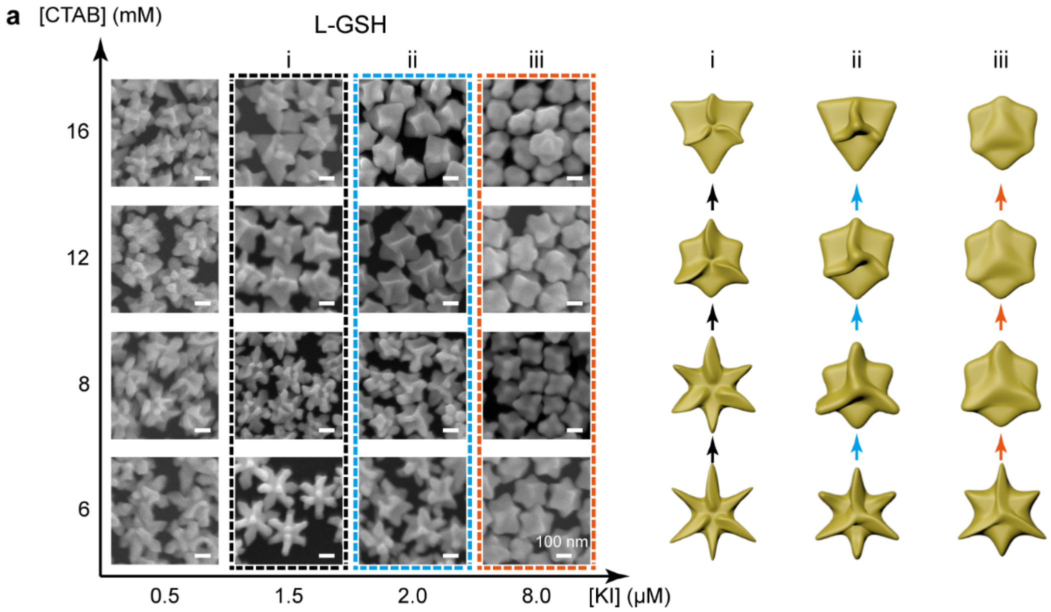

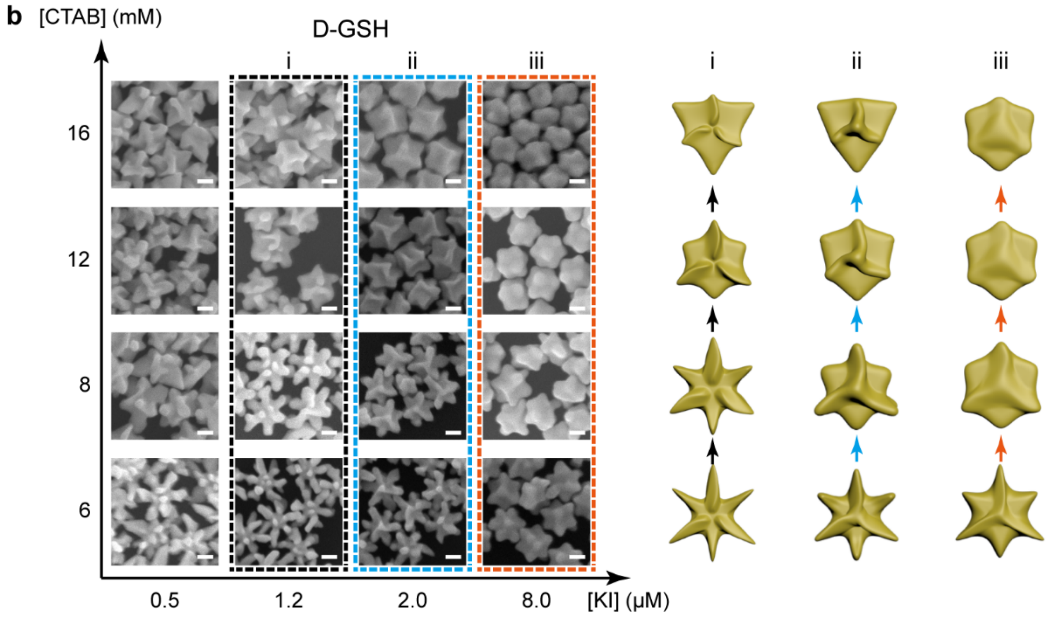

Supplementary Fig. 25 | Effect of increasing CTAB concentrations on the morphology evolution of the Au nanotriskelions through HADG with the 100/30 nm Au nanodisks. a, SEM images and schematics for the nanocrystals synthesized with L-GSH. **b**, SEM images and schematics for the nanocrystals synthesized with D-GSH. All the scale bars are the same.

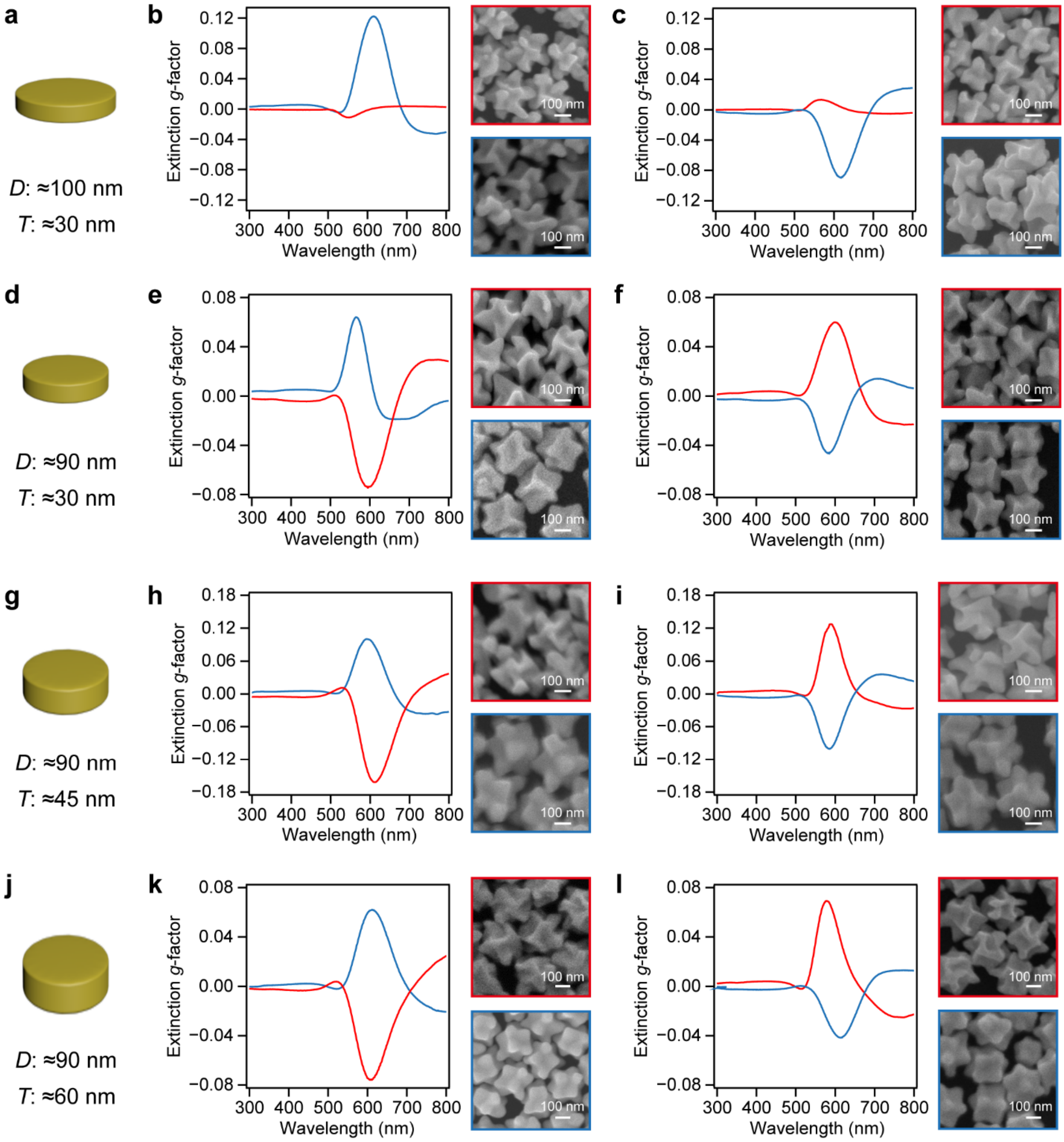

Supplementary Fig. 26 | A library of the Au nanotriskelions and 432 helicoid III nanocrystals prepared through HADG with the Au nanodisks. The Au nanodisks with different diameter-to-thickness aspect ratios, different CTAB/KI concentrations, and GSH enantiomers were employed to synthesize the Au nanotriskelions and 432 helicoid III nanocrystals. **a**–**c**, Using the 100/30 nm Au nanodisks as the seeds. **d**–**f**, Using the 90/30 nm Au nanodisks as the seeds. **g**–**i**, Using the 90/45 nm Au nanodisks as the seeds. **j**–**l**, Using the 90/60 nm Au nanodisks as the seeds. The red plots in the extinction *g*-factor spectra (**b**,**c**,**e**,**f**,**h**,**i**,**k**,**l**) and the SEM images with red boxes (**b**,**c**,**e**,**f**,**h**,**i**,**k**,**l**) show the chirality and structure of the Au nanotriskelions, while the blue ones show the chirality and structure of the 432 helicoid III nanocrystals. The Au nanotriskelions and 432 helicoid III nanocrystals in (**b**,**e**,**h**,**k**) were synthesized from L-GSH while the Au nanotriskelions and 432 helicoid III nanocrystals in (**c**,**f**,**i**,**l**) were synthesized from D-GSH. The 90/45 nm nanodisks can evolve

into nanotriskelions and 432 helicoid III nanocrystals with the absolute values of the extinction g-factors larger than 0.1. Source data are provided as a Source Data file.

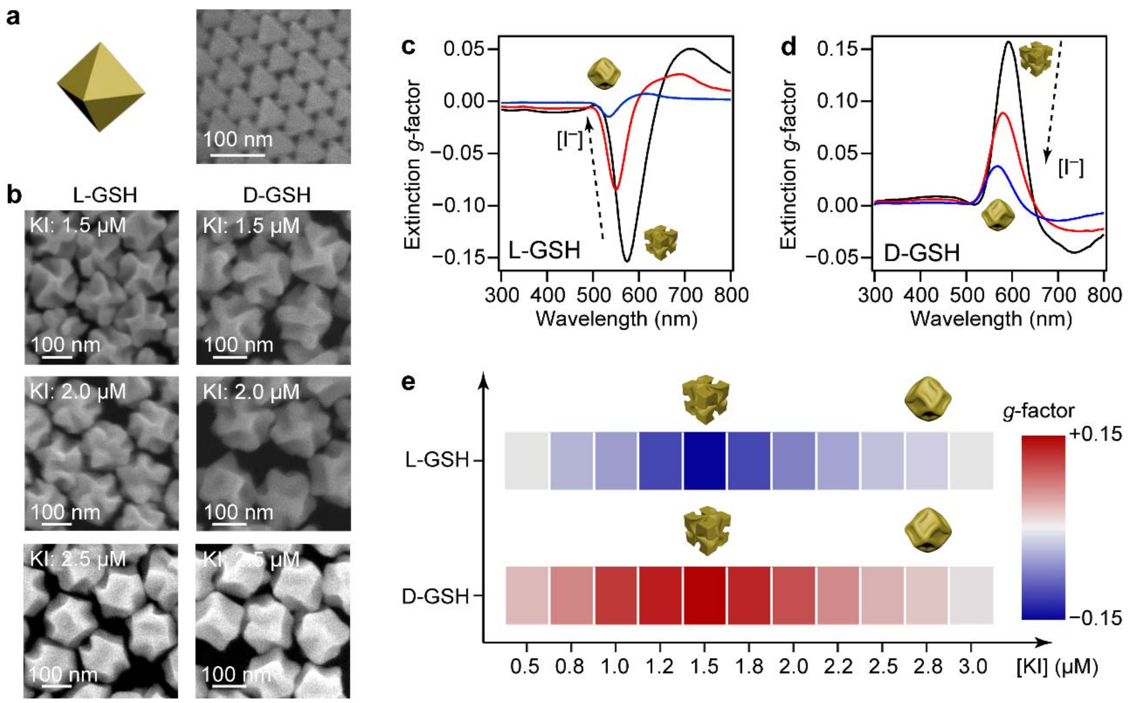

Supplementary Fig. 27 | Morphology and chirality evolution of the chiral nanocrystals grown from Au octahedrons by HADG. a, Model and SEM image of the Au octahedrons with edge lengths of 55 nm. **b**, SEM images showing the effect of the KI concentration on the morphology of the obtained chiral nanocrystals. 432 helicoid III nanocrystals are formed at a low KI concentration (1.5 μM). Further addition of KI finally results in the generation of 432 helicoid IV nanocrystals (KI: 2.5 μM). The morphology evolution was confirmed by SEM imaging. The CTAB concentrations are 10 mM (for L type) and 12 mM (for D type). **c**,**d**, Spectral evolution of the extinction *g*-factors for the chiral nanocrystals in (**b**). The attenuated chirality demonstrates the evolution from the 432 helicoid III to the helicoid IV nanocrystals. The dashed lines show the spectral evolution with increasing KI concentrations. **e**, Dependence of the extinction *g*-factors of the grown chiral nanocrystals on the concentration of KI. Source data are provided as a Source Data file.

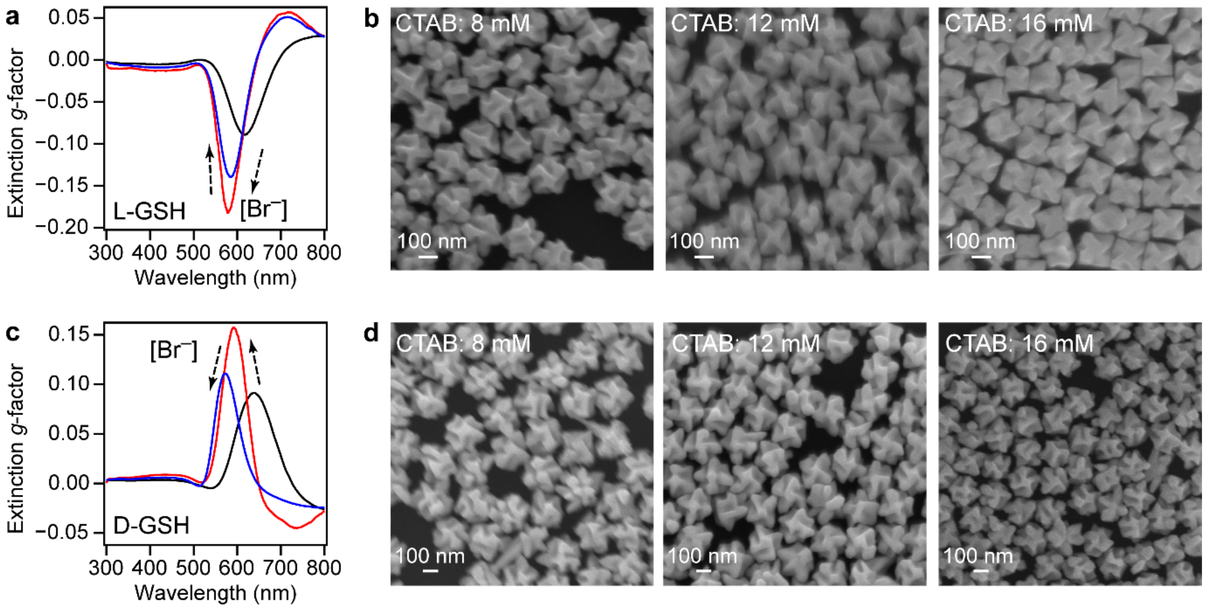

Supplementary Fig. 28 | Effect of the CTAB concentration on the morphology evolution of the 432 helicoid III nanocrystals grown through HADG on the Au octahedrons with edge lengths of 55 nm. a,**b**, Extinction *g*-factor spectra and SEM images of the L-type 432 helicoid III nanocrystals. **c**,**d**, Extinction *g*-factor spectra and SEM images of the D-type 432 helicoid III nanocrystals. The KI concentration was fixed to be 2.0 μM. The twisted surfaces of the 432 helicoid III nanocrystals have no significant change and the 432 helicoid III nanocrystals become more and more cubic with increasing CTAB concentrations. The dashed lines in (**a**,**c**) show the spectral evolution with increasing KI concentrations. Source data are provided as a Source Data file.

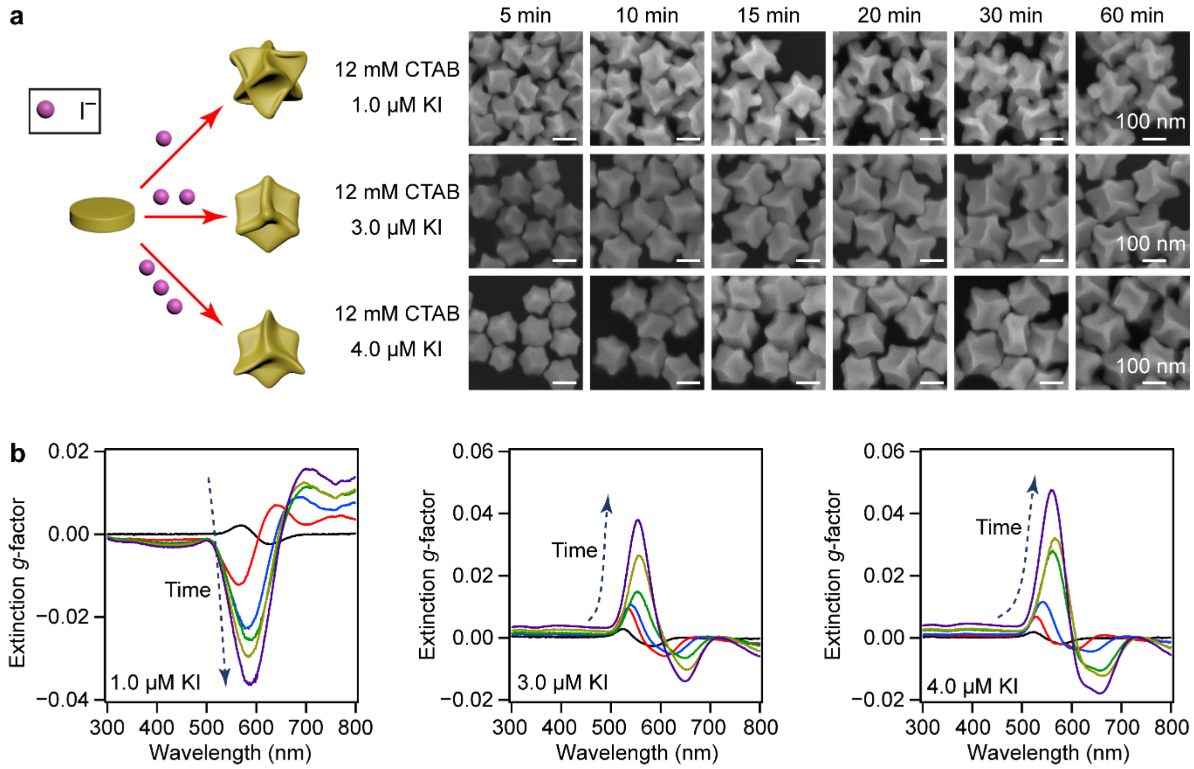

Supplementary Fig. 29 | Time-dependent study of the HADG strategy showing the growth process of the Au nanotriskelions and 432 helicoid III nanocrystals by use of the 90/30 nm Au nanodisks as the seeds. a, Schematic of the growth processes and SEM images of the chiral nanocrystals, showing a sequence of the intermediate morphologies during the evolution. The scale bars in all the SEM images are the same. **b**, Temporal changes in the extinction *g*-factor spectra of the chiral nanocrystals in (**a**). The time-dependent analysis shows that the Au nanodisks first grow into Au nanotriskelions at an early developmental stage (5 min) and then develop into various chiral nanocrystals in an anisotropic manner. The low KI concentration in the growth solution triggers the formation of 432 helicoid III due to the accelerated growth along the <111> directions. The high KI concentration in the growth solution can trigger the formation of Au nanotriskelions by blocking the growth along the <111> directions. The temporal study proved that KI at the increased concentrations can reduce the growth rate along the <111> directions. Source data are provided as a Source Data file.

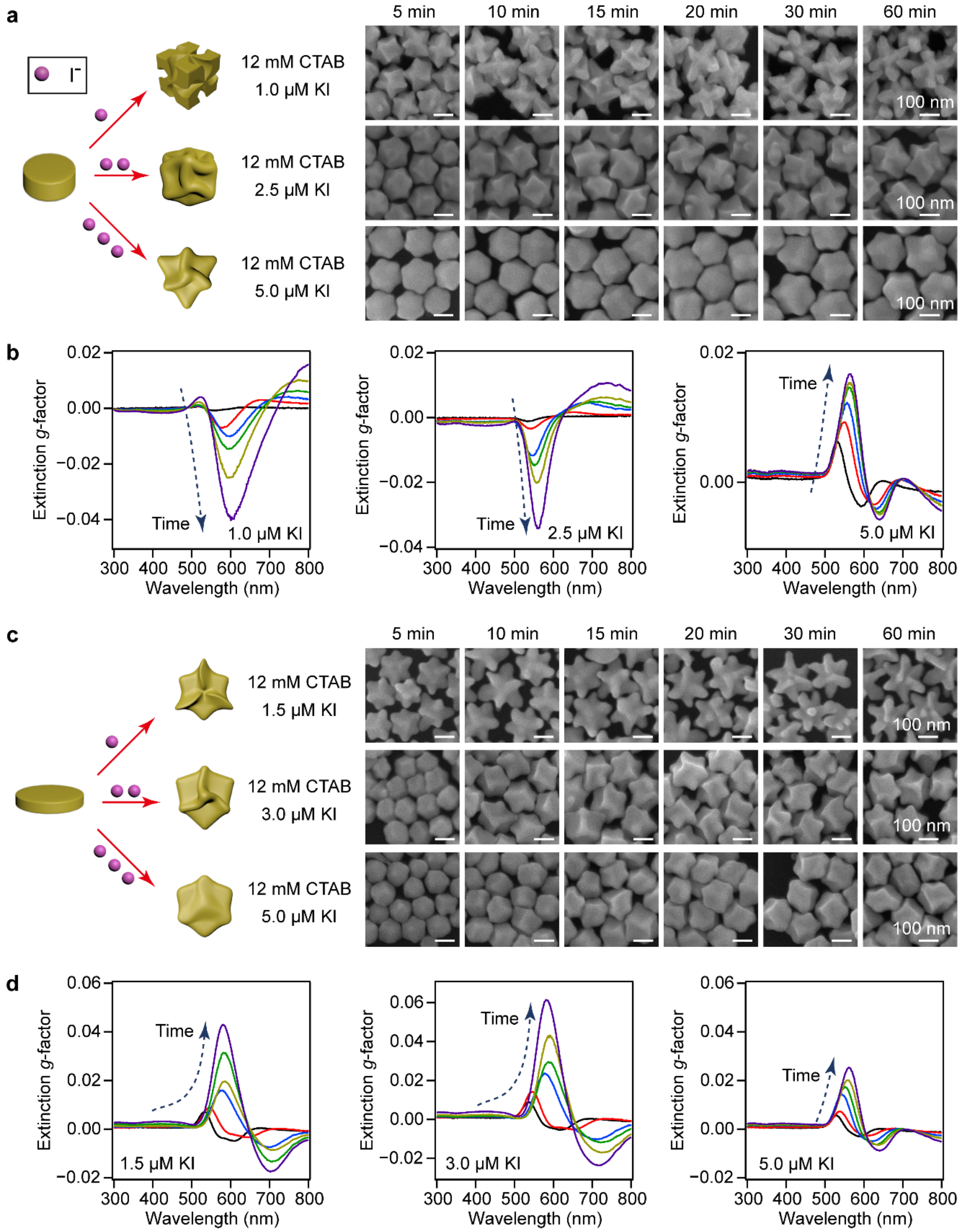

Supplementary Fig. 30 | Chiral nanocrystals synthesized from different nanodisk seeds in the presence of KI with varying concentrations at different time points. a,b, Schematic of the growth processes, SEM images, and extinction *g*-factor spectra of the chiral nanocrystals grown from the 90/60 nm Au nanodisks. **c,d**, Schematic of the growth process, SEM images, and extinction *g*-factor spectra of the chiral nanocrystals grown from the 100/30 nm Au nanodisks. The temporal study proved that KI at the increased concentrations can reduce the growth rate along the <111> directions. All the scale bars in (**a**,**c**) are the same. Source data are provided as a Source Data file.

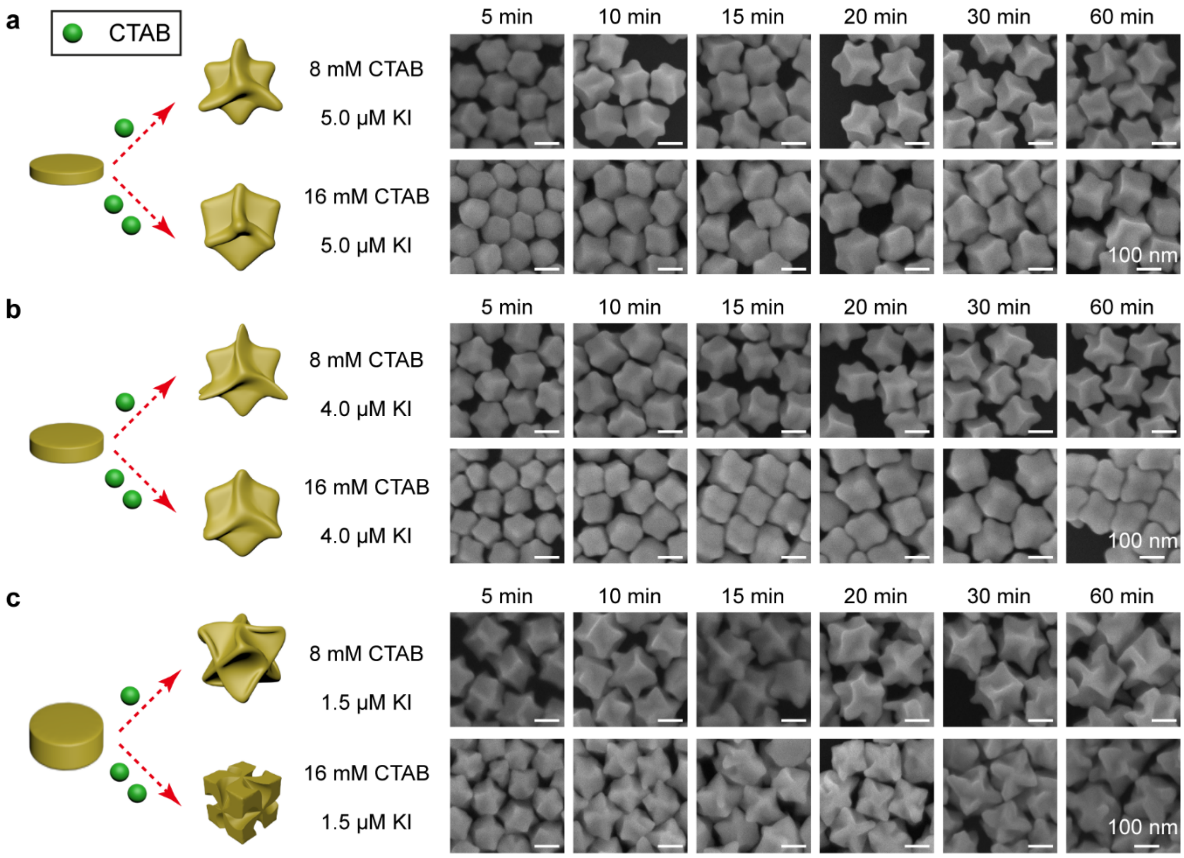

Supplementary Fig. 31 | Chiral nanocrystals synthesized from different Au nanodisk seeds in the presence of CTAB with varying concentrations at different time points. a–c, Schematic of the growth processes and SEM images of the chiral nanocrystals grown from the nanodisks with a diameter/thickness of 100/30 nm (**a**), 90/30 nm (**b**), and 90/60 nm (**c**), respectively. The temporal study proved that CTAB at the increased concentrations can reduce the growth rate along the <100> directions. These observations show that the diverse morphologies are not the result of the enantioselective interaction of the chiral ligands with the chiral facets, but rather of the differential growth along the <100> and <111> directions. The scale bars in all the SEM images are the same.

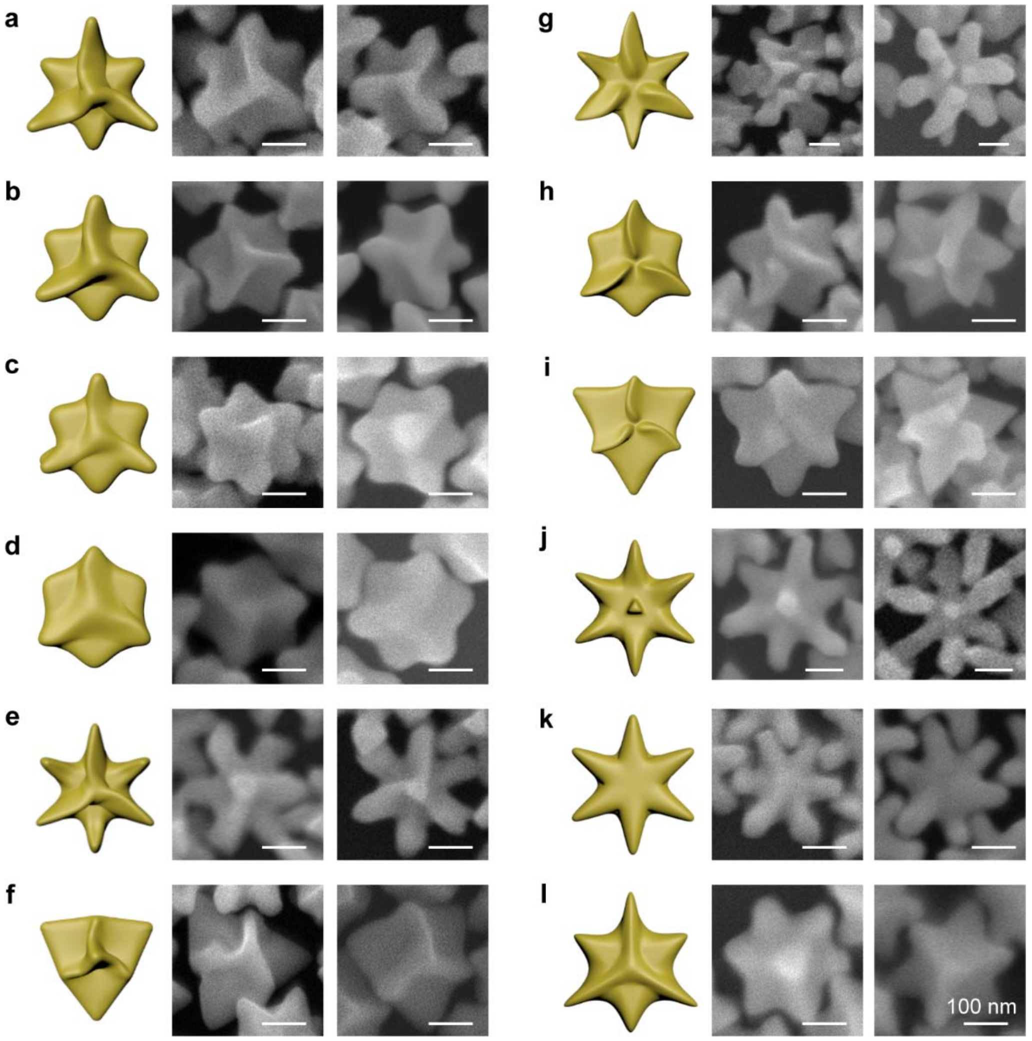

Supplementary Fig. 32 | A library of Au nanotriskelions and nanogears with varying morphologies prepared through HADG with the 100/30 nm nanodisks. Left: models of the L-nanotriskelions. Middle and right: SEM images of the prepared L- and D-nanotriskelions, respectively. The scale bars in all the SEM images are the same. **a**–**d**, Au nanotriskelions synthesized at the same CTAB concentration and different KI concentrations. KI at the increased concentrations blocks the growth along the <111> directions and thus results in the gradual embedment of the twisted arms into the nanodisk domain. **e**,**f**, Au nanotriskelions synthesized at the same KI concentration and different CTAB concentrations. CTAB at the increased concentrations slows the growth rate along the <100> directions and changes the contour of the nanotriskelions from hexagram to triangle. **g**–**i**, Au nanotriskelions with segmented arms synthesized at a fixed low KI concentration and different CTAB concentrations. **j**–**l**, Au nanogears synthesized at a fixed high KI concentration and different CTAB concentrations. KI with high concentrations can help to inhibit the evolution of the chiral surfaces and simultaneously maintain the structure contours. Straight arms are therefore preferentially generated from the {111} facets. The CTAB/KI concentrations for the growth of these nanocrystals are summarized in Supplementary Table 4.

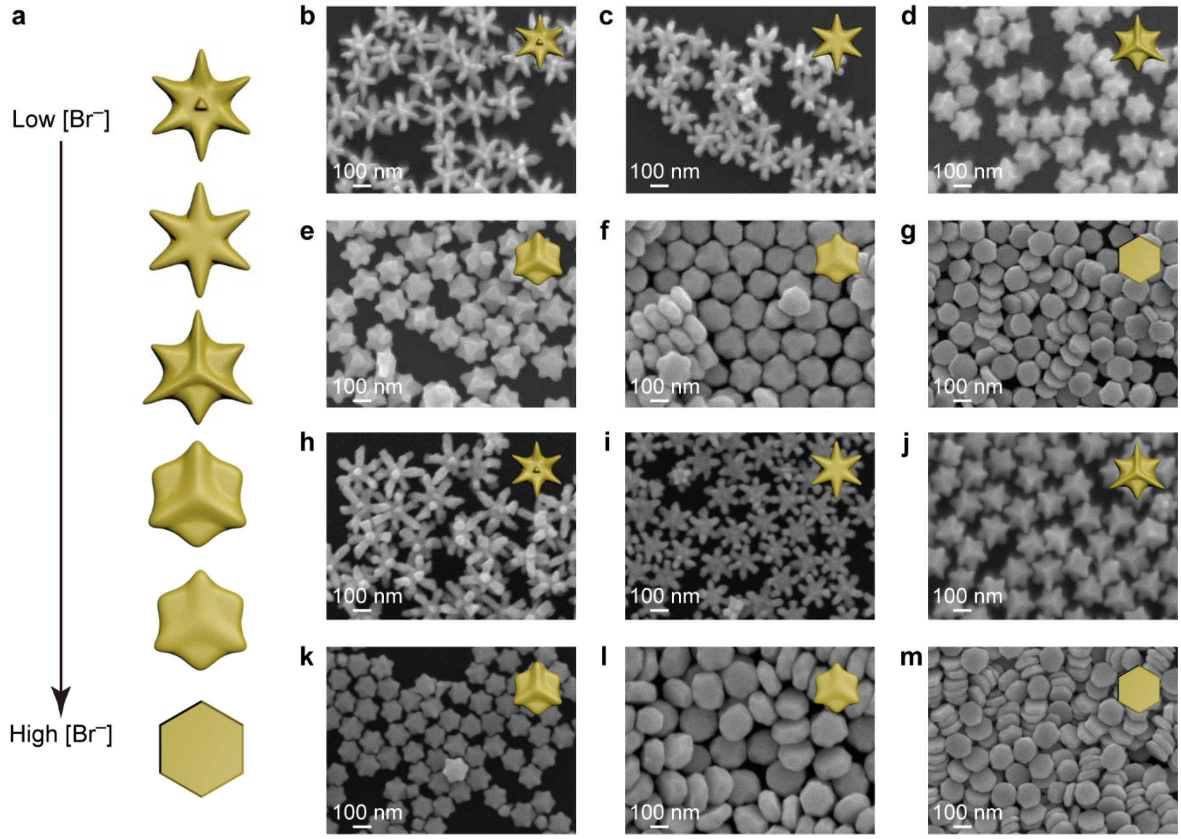

Supplementary Fig. 33 | A library of achiral Au nanocrystals with varying morphologies prepared through HADG on the 100/30 nm nanodisks. KI with a high concentration (10 μM) was employed to inhibit the evolution of the chiral surfaces. **a**, Schematics showing the evolution of the nanocrystal morphology as the CTAB concentration is increased. CTAB at the increased concentrations slows the growth rate along the <100> directions. **b**–**g**, SEM images of the achiral nanocrystals synthesized in the presence of L-GSH. **h**–**m**, SEM images of the achiral nanocrystals synthesized in the presence of D-GSH. The CTAB/KI concentrations for these nanocrystals are summarized in Supplementary Table 4.

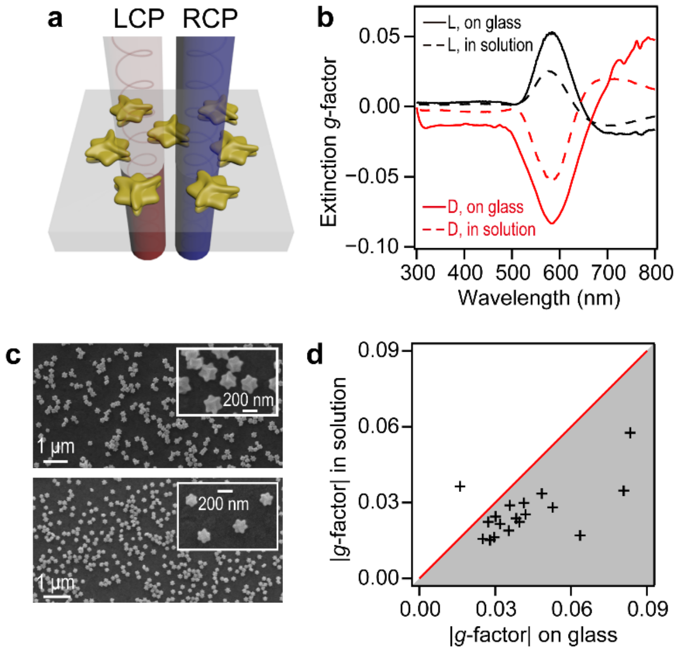

Supplementary Fig. 34 | Gold nanotriskelion arrays on substrates. a, Schematic showing the chiroptical response of the Au nanotriskelion-deposited substrate. **b**, Extinction *g*-factor spectra of the Au nanotriskelions with different geometric handedness deposited on silica substrates and dispersed in solution. The Au nanotriskelion-based substrates were immersed in water for the spectral measurements. **c**, SEM images of the Au nanotriskelion-deposited substrates in (**b**). Top: L-nanotriskelions. Bottom: D-nanotriskelions. The insets show the zoomed-in SEM images. Over 85% of the Au nanotriskelions face upward when deposited on the substrates. **d**, Comparison of the extinction *g*-factors for the Au nanotriskelions deposited on silica substrates and dispersed in solution. The CD intensities can be different because of the absorption difference between the solution and the substrate. We therefore calculated the *g*-factors to eliminate the influence of the absorption difference. The chiroptical response of the colloidal nanocrystals dispersed in solution is weaker than that of the same nanocrystals deposited on the substrates, which results from the random orientation of the nanocrystals in solution. Source data are provided as a Source Data file.

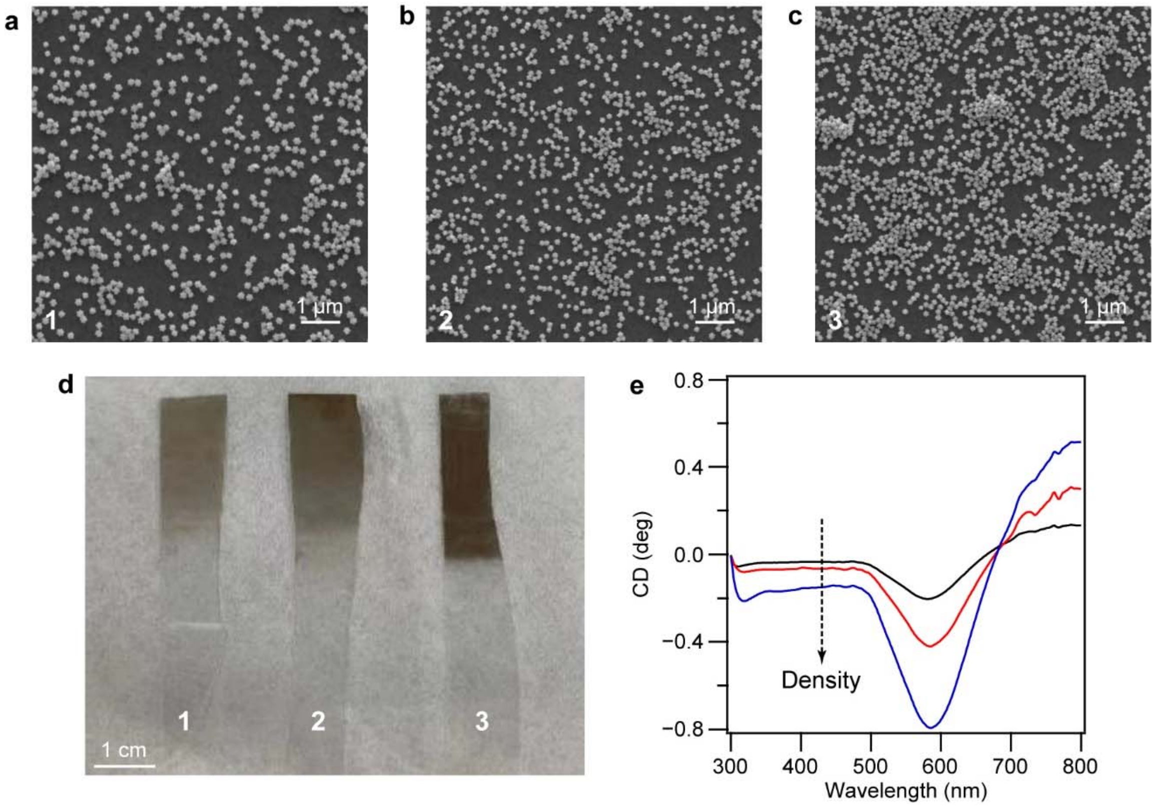

Supplementary Fig. 35 | Au nanotriskelion-deposited substrates with varying particle densities and CD response. a–**c**, SEM images of the Au nanotriskelion-deposited substrates with increased particle densities. The nanocrystal density can be controlled by the deposition time. **d**, Photograph of the glass substrates deposited with the Au nanotriskelions of increasing particle densities. Increasing color contrast can be observed as the nanocrystal number density is increased. **e**, CD spectra of the substrates in (**a**–**c**). The substrates were immersed in water for the CD measurements. The dashed lines show the spectral evolution with increasing particle densities. Enhanced chiral response was observed with increasing nanocrystal number densities. Source data are provided as a Source Data file.

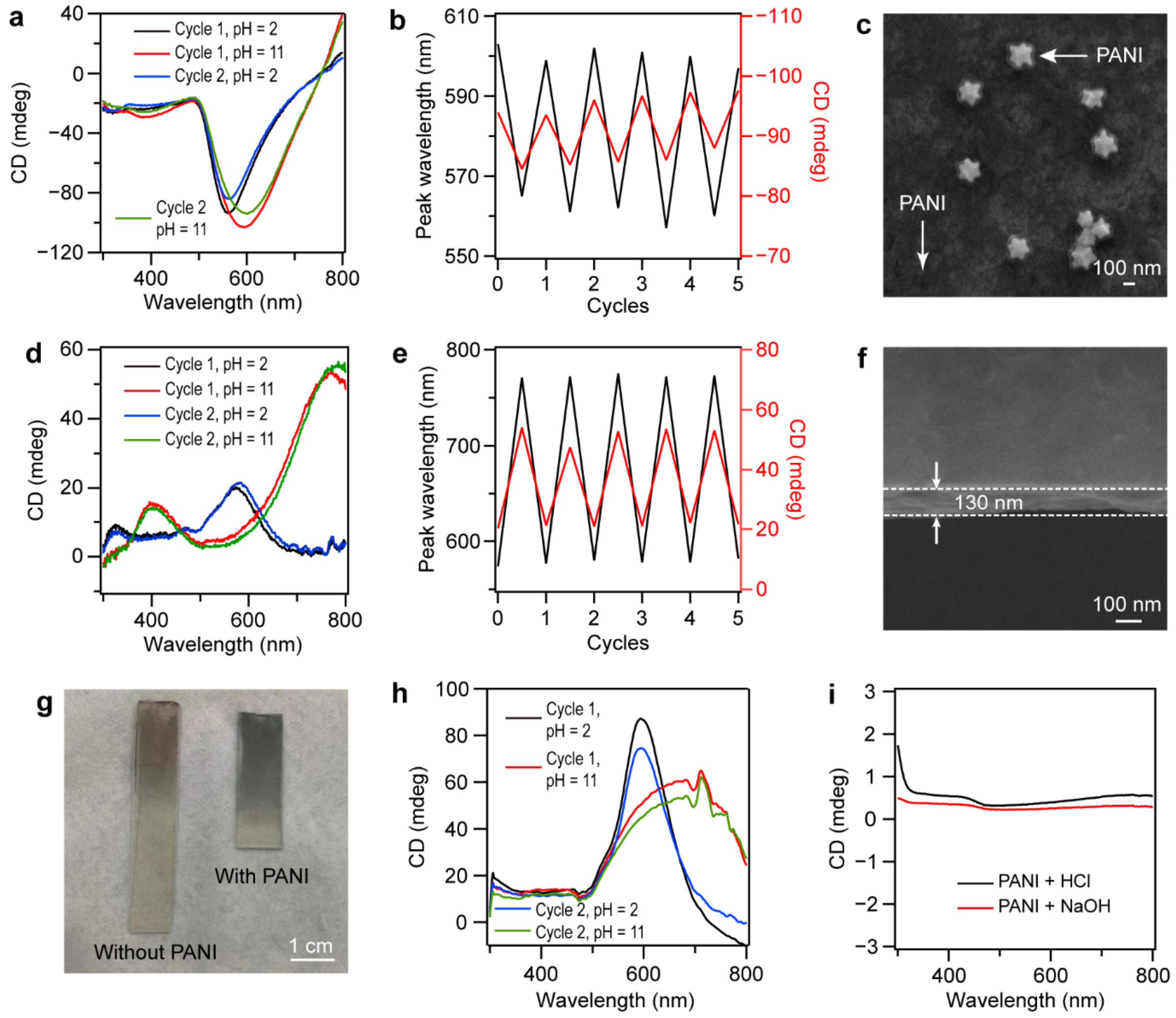

Supplementary Fig. 36 | Switching of the chiroptical response of the Au nanotriskelion/PANI substrate. a–c, Switching of the D-nanotriskelion-deposited substrate covered with a 20 nm PANI layer. The CD spectra during the proton-doping and dedoping processes in 2 cycles (**a**) and the modulation of the peak wavelength and CD value over 5 cycles (**b**) are provided. The SEM image (**c**) clearly shows the successful deposition of the 20 nm PANI layer. **d–f**, Switching of the L-nanotriskelion-deposited substrate covered with a 130 nm PANI layer. Peak shifts as large as 200 nm were achieved over 5 cycles of switching. **g**, Photograph of an L-nanotriskelion-deposited substrate before and after coating with the PANI layer through electrochemical deposition. **h**, CD spectra of the chiral substrate in (**g**) during the proton-doping and dedoping processes in 2 cycles. **i**, CD spectra of a clean glass substrate coated with PANI. CD signal can hardly be detected, indicating the achiral response of the PANI layer. Source data are provided as a Source Data file.

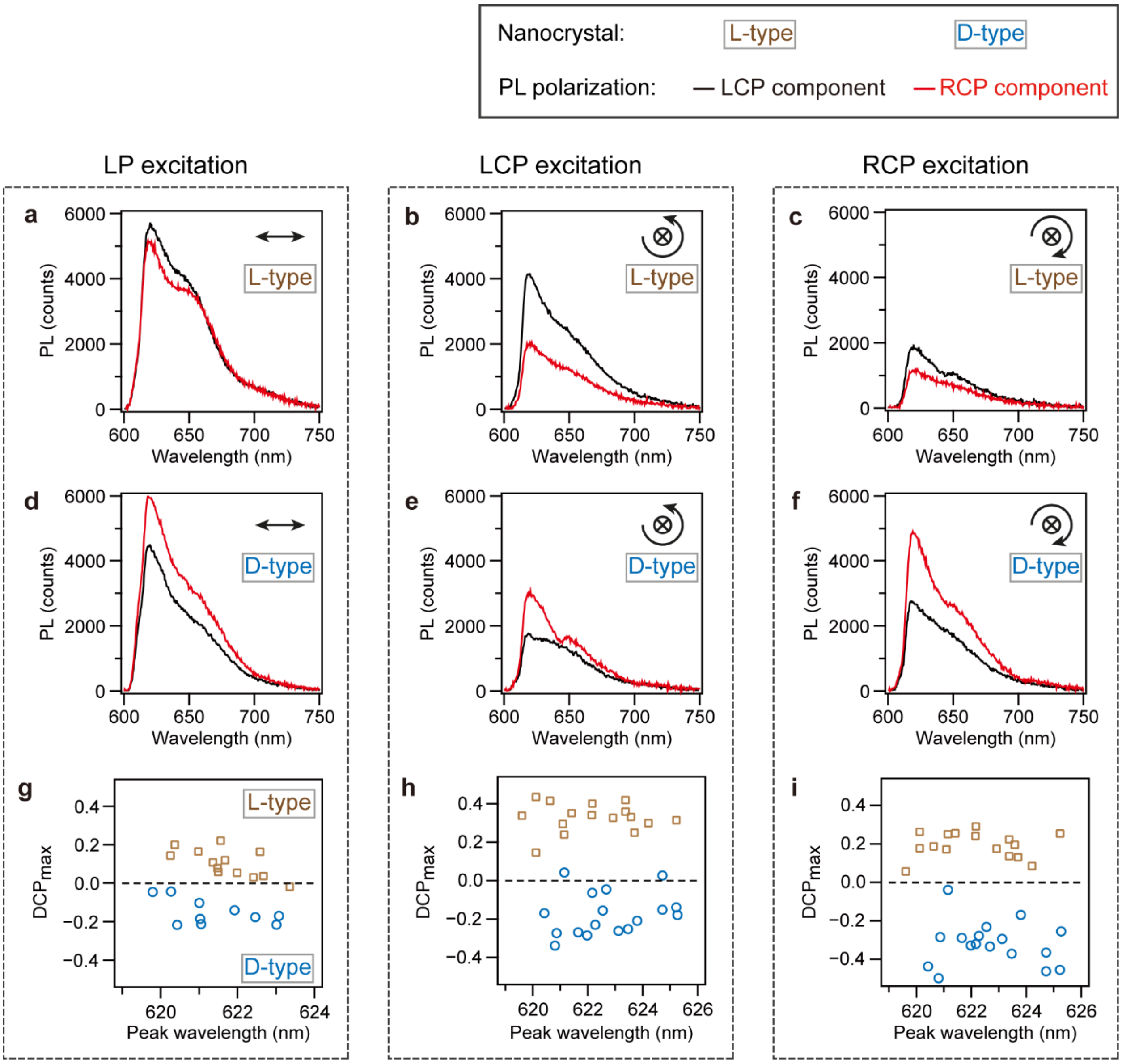

Supplementary Fig. 37 | Polarization-resolved PL spectra from the hybrid nanostructures made of the L- and D-type chiral nanotriskelions under the excitation of differently polarized laser light. a–c, L-type nanostructures excited by LP (**a**), LCP (**b**), and RCP laser light (**c**). **d–f**, D-type nanostructures excited by LP (**d**), LCP (**e**), and RCP laser light (**f**). **g–i**, Maximal DCP values and peak wavelengths for the different L-type and D-type nanostructures under excitation of LP (**g**), LCP (**h**), and RCP laser light (**i**). Source data are provided as a Source Data file.

Supplementary Table 1 | LSPR peak wavelengths and dimensions of the Au nanodisk samples.

Au nanodisks	LSPR wavelength	Diameter (nm)	Thickness (nm)
75/30	590 nm	74.8 ± 8.4	29.5 ± 4.3
90/30	600 nm	90.7 ± 9.1	29.9 ± 4.7
100/30	615 nm	100.6 ± 12.0	30.3 ± 4.8
120/30	628 nm	121.8 ± 13.7	29.8 ± 4.6
90/20	640 nm	90.2 ± 13.5	19.3 ± 3.6
90/45	580 nm	91.3 ± 10.8	45.2 ± 6.0
90/60	560 nm	89.1 ± 10.0	58.7 ± 8.8

Supplementary Table 2 | Parameters of the Au nanotriskelions in Fig. 3 and Supplementary Fig. 14, including the sizes, CD properties, and used CTAB/KI concentrations for the growth of the nanotriskelions.

Nanotriskelions	GSH	Length (nm)	Thickness (nm)	*g*-factor	CD peak	CTAB (mM)	KI (μM)	$HAuCl_4$ (μmol)
90/30-1.5	L	93.2 ± 8.7	109.7	0.0095	548 nm	8	1.5	1.5
90/30-3	L	109.8 ± 7.5	127.7	0.042	562 nm	8	2.0	3
90/30-4	L	119.0 ± 9.9	140.2	0.064	566 nm	8	2.5	4
90/30-5	L	132.8 ± 9.6	154.2	0.083	584 nm	8	3.0	5
90/20-4	L	95.1 ± 8.4	87.6	0.013	543 nm	8	1.5	4
90/45-4	L	132.5 ± 8.4	193.0	0.10	590 nm	12	4.0	4
90/60-4	L	139.7 ± 7.8	232.5	0.062	614 nm	16	4.0	4
75/30-4	L	109.0 ± 8.3	143.2	0.015	549 nm	12	3.0	4
100/30-4	L	149.6 ± 10.3	141.2	0.12	616 nm	8	2.0	4
120/30-4	L	159.9 ± 14.8	148.6	0.061	645 nm	12	1.5	4
90/30-1.5	D	96.2 ± 6.6	112.4	-0.014	547 nm	8	1.5	1.5
90/30-3	D	111.7 ± 8.8	125.7	-0.039	566 nm	8	2.0	3
90/30-4	D	125.2 ± 10.0	144.1	-0.046	586 nm	8	2.0	4
90/30-5	D	136.0 ± 11.3	160.7	-0.080	594 nm	8	3.0	5
90/20-4	D	93.4 ± 5.8	87.4	-0.014	534 nm	8	1.0	4
90/45-4	D	133.3 ± 8.1	189.0	-0.10	586 nm	12	4.0	4
90/60-4	D	141.0 ± 9.3	225.0	-0.039	611 nm	12	8.0	4
75/30-4	D	111.6 ± 7.3	150.6	-0.020	542 nm	12	2.5	4
100/30-4	D	149.0 ± 12.0	148.5	-0.090	618 nm	8	1.8	4
120/30-4	D	159.4 ± 10.5	150.6	-0.054	642 nm	12	1.5	4

Supplementary Table 3 | Parameters of the chiral nanocrystals in Supplementary Fig. 26, including the CD properties and used CTAB/KI concentrations during growth and the dominant rotational symmetry.

Seeds	GSH	Extinction *g*-factor	Peak wavelength	CTAB (mM)	KI (μM)	Dominant rotational symmetry
100/30	L	0.12	616 nm	8	2.0	TFR
100/30	L	-0.011	556 nm	16	0.5	FFR
90/30	L	0.064	566 nm	8	2.5	TFR
90/30	L	-0.074	591 nm	12	0.5	FFR
90/45	L	0.10	590 nm	12	4.0	TFR
90/45	L	-0.16	610 nm	8	1.2	FFR
90/60	L	0.062	614 nm	16	4.0	TFR
90/60	L	-0.076	606 nm	12	1.5	FFR
100/30	D	-0.090	618 nm	8	1.8	TFR
100/30	D	0.013	568 nm	16	0.5	FFR
90/30	D	-0.046	586 nm	8	2.0	TFR
90/30	D	0.060	600 nm	12	0.5	FFR
90/45	D	-0.10	586 nm	12	4.0	TFR
90/45	D	0.13	590 nm	8	1.2	FFR
90/60	D	-0.039	611 nm	12	8.0	TFR
90/60	D	0.079	579 nm	12	1.5	FFR

Supplementary Table 4 | Growth conditions of the Au nanotriskelions in Figs. 4, 5, Supplementary Figs. 8, 17, 20, 22, 24, 32, and 33, including the seeds, chiral ligands, and used CTAB/KI concentrations.

Related figure	Seeds	Ligand	CTAB (mM)	KI (μM)
Fig. 4a, b	90/30	L-GSH	8	0.5, 1.0, 1.2, 1.5, and 2.5
Fig. 4a, b	90/30	D-GSH	8	0.5, 1.0, 1.2, 1.5, and 2.0
Fig. 5b	90/30	D-GSH	12	2.5
Fig. 5e	90/30	L-GSH	8	2.5
Fig. 5e	90/30	D-GSH	8	2.0
Supplementary Fig. 8	90/30	L-Cys	8	3.0
Supplementary Fig. 8	90/30	D-Cys	8	3.0
Supplementary Fig. 17a	90/30	L-GSH	8	2.0
Supplementary Fig. 17b	90/30	L-GSH	8	1.8
Supplementary Fig. 17d	90/30	D-GSH	8	2.5
Supplementary Fig. 17e	90/30	D-GSH	8	2.0
Supplementary Fig. 20a, b	90/45	L-GSH	12	1.2, 1.5, 1.8, 3.0, and 4.0
Supplementary Fig. 20a, b	90/45	D-GSH	12	1.2, 1.5, 2.0, 3.0, and 4.0
Supplementary Fig. 22a, b	90/60	L-GSH	12	1.5, 2.5, 3.0, 4.0, and 5.0
Supplementary Fig. 22a, b	90/60	D-GSH	12	1.5, 2.0, 2.5, 5.0, and 8.0
Supplementary Fig. 24a, b	100/30	L-GSH	8	1.5, 1.8, 2.0, 2.5, and 8.0
Supplementary Fig. 24a, b	100/30	D-GSH	8	1.2, 1.5, 1.8, 3.0, and 8.0

Supplementary Fig. 32a–d	100/30	L-GSH	8	1.8, 2.0, 2.5, and 8.0
Supplementary Fig. 32a–d	100/30	D-GSH	8	1.5, 1.8, 3.0, and 8.0
Supplementary Fig. 32e, f	100/30	L-GSH	6 and 16	2.0
Supplementary Fig. 32e, f	100/30	D-GSH	6 and 16	2.0
Supplementary Fig. 32g–i	100/30	L-GSH	8, 12, and 16	1.5
Supplementary Fig. 32g–i	100/30	D-GSH	8, 12, and 16	1.2
Supplementary Fig. 32j–l	100/30	L-GSH	2, 4, and 6	10
Supplementary Fig. 32j–l	100/30	D-GSH	2, 4, and 6	10
Supplementary Fig. 33b–g	100/30	L-GSH	2, 4, 6, 8, 12, and 16	10
Supplementary Fig. 33h–m	100/30	D-GSH	2, 4, 6, 8, 12, and 16	10

Supplementary references

1. Jiang, Q. N. et al. Synthesis and high electrocatalytic performance of hexagram shaped gold particles having an open surface structure with kinks. *Nano Res.* **4**, 612–622 (2011).
2. Grzelczak, M. et al. Shape control in gold nanoparticle synthesis. *Chem. Soc. Rev.* **37**, 1783–1791 (2008).
