# Supplementary material for: Halide-assisted differential growth of chiral nanoparticles with threefold rotational symmetry
Source: Nat Commun. 2023 Jun 24;14:3783. doi: 10.1038/s41467-023-39456-8 (PMC10290678; doi:10.1038/s41467-023-39456-8)
Supplement: Supplementary file 3 — Description of additional supplementary files [file 41467_2023_39456_MOESM3_ESM.pdf]

<u>Description of additional supplementary files</u>

Supplementary Movie 1 : Tomography reconstruction (left) and constructed model (right) of the Lnanotriskelions.

Supplementary Movie 2 : Tomography reconstruction (left) and constructed model (right) of the Dnanotriskelions.
